# Quercetin as a Therapeutic Option in a Rat Model of Aluminum Chloride- and D-Galactose-Induced Neurodegeneration

**DOI:** 10.3390/ijms26125743

**Published:** 2025-06-15

**Authors:** Marina Kukolj, Nada Oršolić, Lea Langer Horvat, Barbara Nikolić, Tatjana Ocrt, Karmen Branović Čakanić, Romana Gračan, Ivana Zrinščak, Maja Jazvinšćak Jembrek, Goran Šimić

**Affiliations:** 1 Division of Animal Physiology, Faculty of Science, University of Zagreb, Rooseveltov trg 6, 10000 Zagreb, Croatia; kukoljmarina@gmail.com (M.K.); barbara.nikolic@biol.pmf.hr (B.N.); romana.gracan@biol.pmf.hr (R.G.); 2 Department of Neuroscience, Croatian Institute for Brain Research, School of Medicine, University of Zagreb, Šalata 12, 10000 Zagreb, Croatia; lea.langer@yahoo.co.uk (L.L.H.); gsimic@hiim.hr (G.Š.); 3 Analytical Toxicilogy and Mineral Metabolism Unit, Institute for Medical Research and Occupational Health, 10000 Zagreb, Croatia; 4 Croatian Veterinary Institute, Savska cesta 143, 10000 Zagreb, Croatia; branovic@veinst.hr; 5 Ministry of the Interior, Varaždin Regional Civil Protection Office, Kratka 1/IV, 42000 Varaždin, Croatia; zrinscak.ivana@gmail.com; 6 Division of Molecular Medicine, Laboratory for Protein Dynamics, Ruđer Bošković Institute, Bijenička cesta 54, 10000 Zagreb, Croatia; maja.jazvinscak.jembrek@irb.hr; 7 School of Medicine, Catholic University of Croatia, Ilica 244, 10000 Zagreb, Croatia

**Keywords:** aluminum/D-galactose-induced Alzheimer’s disease, metal disbalance, oxidative-inflammatory markers, neurodegeneration, quercetin, cellular and molecular neuroprotective effects of quercetin, molecular insight

## Abstract

Aluminum (Al) is one of the most abundant metals on Earth and is well known as an environmental neurotoxic agent in the pathogenesis of Alzheimer’s disease. Aluminum toxicity is associated with oxidative stress, reduction of antioxidant enzymes, and disruption of the balance of cellular metals, such as iron (Fe), calcium (Ca), and copper (Cu), which causes structural and functional changes in the nervous tissue of the brain or peripheral nervous system. The intake of functional foods, rich in antioxidants, such as quercetin, may be beneficial in combating oxidative stress and neurodegenerative changes in the brain. The aim of this study was to provide deeper insight into the cellular and molecular neuroprotective effects of quercetin in regulating amyloid-beta (Aβ) accumulation, tau pathology, and neuroinflammation in the Al/D-galactose-induced rat model (Al/D-gal) of AD. The results showed that quercetin successfully modulated the impaired homeostatic and neuropathological consequences of aluminum chloride and D-galactose administration over 28 days: it directly protected neurons by regulating the level of oxidative stress and antioxidants, reduced Aβ aggregation by inhibiting the activity of acetylcholinesterase (AChE), increased the survival, growth, and differentiation of nerve cells by maintaining the level of brain-derived neurotrophic factor (BDNF), and regulated microglial immunoreactivity and neuroinflammation by reducing the level of proinflammatory cytokines. The multiple effects confirm that quercetin can be applied as an alternative non-pharmaceutical approach in reducing Al-induced neurotoxicity and maintaining adaptive homeostasis, which consequently affects the functioning of the central nervous system and the whole organism.

## 1. Introduction

Alzheimer’s disease (AD) is one of the most common neurodegenerative disorders. It is characterized by the progressive loss of central nervous system (CNS) cells, leading to the dysfunction and deterioration of brain function. This results in cognitive impairment and behavioral changes [1]. The first symptoms typically appear after the fourth or fifth decade of life due to the disease’s slow progression [2]. In recent years, an increase in life expectancy has resulted in a rising prevalence of neurodegenerative diseases, with up to 2% of the population in developed countries affected at any given time [3]. The growing number of AD patients represents a significant social and economic burden, with projections estimating that the number of patients will reach 131.5 million people by 2050 [4,5].

AD primarily affects synapses and neurons in the cortical and limbic structures of the brain, including the hippocampus and amygdala [1,4,5], and most importantly the lateral entorhinal cortex, which is likely the earliest site affected [6]. Several hypotheses have been proposed to explain the origin of AD pathology. The most extensively studied are the amyloid-β peptide (Aβ) intra- and extracellular accumulation and hyperphosphorylated tau (p-tau) protein hypotheses. Aβ aggregation results in the formation of extracellular amyloid plaques (APs), while abnormal deposits of p-tau proteins form neurofibrillary tangles (NFTs). Other hypotheses include oxidative stress (OS), the mitochondrial cascade hypothesis, the cholinergic hypothesis, and neuroinflammation [7]. Mitochondrial dysfunction, hormonal imbalance, synaptic damage, impaired neurotransmission, inflammatory responses and increased pro-inflammatory signaling, changes in innate and adaptive immune signaling, and cell cycle deregulation all contribute to AD pathology [8]. These mechanisms are closely related to progressive dementia and the loss of cognitive functions [7,8]. The most significant risk factor for AD is aging, while multiple genetic and environmental factors also play important roles in disease progression [4,5,6,7,8].

Aluminum (Al) is one of the most abundant metals in the environment. It exists in only one oxidation state, Al^3+^, and shows strong affinity for negatively charged, oxygen-donor ligands. In the brain, Al induces OS by disrupting the balance between oxidants and antioxidants and interfering with the normal function of mitochondria and various enzymes [9]. It can induce oxidative damage through the production of reactive radicals by multiple mechanisms. Due to its positive charge, Al^3+^ can bind to negatively charged brain phospholipids, which contain polyunsaturated fatty acids that are highly susceptible to attack by reactive oxygen species (ROS), such as superoxide anion (O_2_˙^−^), hydrogen peroxide (H_2_O_2_), hydroxyl radical (OH˙), and hydroxyl anion (OH^−^). Furthermore, Al^3+^ binds to metal-binding amino acids, like histidine, arginine, and tyrosine, or to phosphorylated amino acids, linking them together and promoting the aggregation of highly phosphorylated cytoskeletal proteins, such as neurofilaments and microtubule-associated proteins (MAPs) [9,10,11]. In addition, aggregated Aβ can further increase free radical production and cause additional brain lesions [9,10,11]. Moreover, aluminum can promote the Fenton reaction by disrupting iron homeostasis, leading to iron-mediated lipid peroxidation (LPO) and the generation of highly reactive radicals, such as hydroxyl radicals, along with the accumulation of Fe^3+^.

Al^3+^ binds to O_2_˙^−^ to form an Al-O_2_˙^−^ complex, which increases the oxidative capacity of O_2_˙^−^. Al^3+^ also binds to the phosphate groups of DNA and RNA, altering DNA topology and influencing the expression of genes essential for brain function [9,10,11]. Furthermore, Al impairs the activity of various enzymes, inhibits voltage-gated calcium (Ca^2+^) channels and neurotransmitter receptors, and disrupts synaptic transmission, ultimately leading to the apoptotic death of neurons and glial cells. Among the toxic effects of Al, cytotoxicity mediated by free radicals, LPO, and alterations in essential elements in both the brain and serum have been observed [9,10,11,12,13].

Chronic administration of D-galactose (D-gal), a reducing sugar, at low doses influences the natural aging process in animals, leading to OS, cognitive impairment, reduced immune response, and changes in gene transcription [11]. In addition, D-galactose initiates non-enzymatic glycation reactions with the free amines of amino acids in peptides and proteins that ultimately result in the formation of advanced glycation end products (AGEs). AGEs then interact with their receptors (RAGE) and produce ROS. ROS increases DNA fragmentation and cell death by endonucleases in the dentate gyrus (DG) and in the CA1 and CA3 regions of the hippocampus. These characteristics suggest that a combination of AlCl_3_ and D-galactose (Al/D-gal) serves as an effective senescence model, inducing OS and neurotoxicity, two important hallmarks of AD. Neuronal damage induced by AlCl_3_ and D-galactose is thus a valuable model for studying the mechanisms of AD and for screening potential therapeutic drugs [11].

Numerous studies have demonstrated that natural herbal remedies and a flavonoid-rich diet can improve mental health and cognitive functions. Quercetin has gained much attention for its potential therapeutic properties, including anti-cancinogenic, antiviral anti-allergic, anti-diabetic, antithrombotic, anti-ischemic, and anti-inflammatory effects. It also shows protective effects in neurodegenerative diseases, atherosclerosis, and coronary heart disease. Because OS is implicated in the pathogenesis of these conditions, the antioxidant activity of quercetin may be at least partly responsible for its protective effects. Quercetin is one of the most powerful natural antioxidants and has been reported to scavenge O_2_˙^−^, singlet oxygen (^1^O_2_), and hydroxyl radicals. It also inhibits LPO, suppresses cyclooxygenase and lipoxygenase enzymes, and chelates transition metal ions, such as Fe and Cu [14]. Several studies suggest that quercetin has anti-amyloidogenic properties and exerts neuroprotective effects against neurological diseases [15,16,17,18]. Quercetin-3-O-glucuronide (Q3GA), a major metabolite present in human and rat plasma, can cross the BBB and the blood–cerebrospinal fluid barrier (BCSFB). It accumulates in specific cell types, such as macrophages, where it is deconjugated into bioactive aglycone forms, retaining potent antioxidant and anti-inflammatory activities [16]. However, previous research has primarily focused on behavioral changes in animal models without investigating cellular and molecular alterations in the brain [11]. Currently, there is limited information regarding the specific biological activities and cellular/molecular mechanisms by which quercetin may beneficially modulate AD-related neuropathological changes and cognitive dysfunction.

The aim of this study was to provide deeper insight into the cellular and molecular neuroprotective effects of quercetin in regulating Aβ accumulation, tau pathology, and neuroinflammation in the Al/D-gal-induced model of AD. Specifically, we sought to establish a link between the antioxidant activity and metal-chelating properties of quercetin and the Al/D-gal-induced OS and accumulation of Fe, Al, and Ca in the brain and serum, as well as to evaluate its effect on the development of neuropathological changes in the brain. Histological analysis was conducted using hematoxylin-eosin staining and the modified Bielschowsky method, and immunohistochemical labeling of key markers, including Aβ (4G8), neurofibrillary tangles (AT8, PHF1, and MC1), and microglia (Iba1, CD68, and HLA-DR) in the brain. Furthermore, we aimed to assess the potential efficacy of quercetin in mitigating Al/D-gal-induced neuroinflammation by monitoring pro-inflammatory cytokine levels and changes in BDNF and AChE activity. The findings of this study contribute to a better understanding of the neuroprotective potential of quercetin in preventing neurodegenerative damage induced by environmental factors, such as aluminum.

## 2. Results

### 2.1. The Effect of Chronic Administration of Al/D-Gal and Quercetin on Body Weight in Experimental Animals

**Figure 1:**
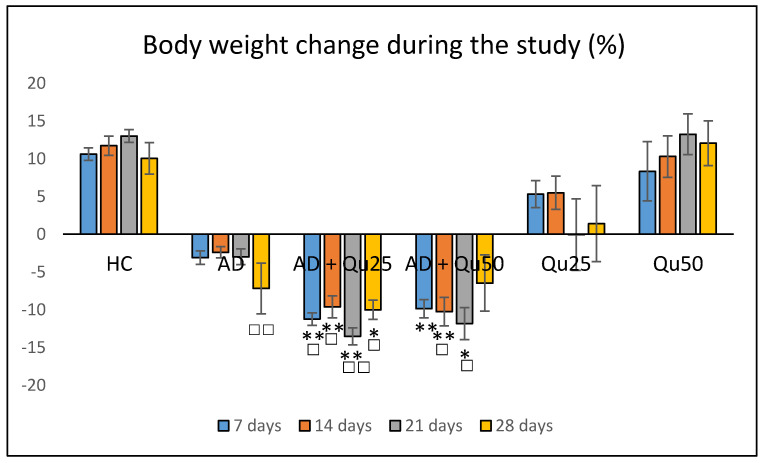
Body weight changes in rats following chronic administration of aluminum chloride + D-galactose and quercetin. Rats (n = 10 per group) were injected with aluminum chloride + D-galactose (10 mg/kg + 60 mg/kg) and quercetin at doses of 25 or 50 mg/kg through the intraperitoneal route (*i.p.*) for 28 days. Control rats received intraperitoneal saline solution. Data are presented as the mean ± SEM and were analyzed using the Kruskal–Wallis nonparametric test. * Significantly different compared to the healthy control (HC) group (* *p* ≤ 0.05; ** *p* ≤ 0.01). ^□^ Significantly different compared to the Qu_50_ group (^□^ *p* ≤ 0.05; ^□□^ *p* ≤ 0.01). Abbreviations: HC—Healthy control group; AD—Alzheimer’s disease rat model; AD + Qu_25_—AD model injected with quercetin *i.p.* at a dose of 25 mg/kg; AD + Qu_50_—AD model injected with quercetin *i.p.* at a dose of 50 mg/kg; SEM—standard error of the mean.

Aluminum has no biological role and is classified as a non-essential toxic metal that affects metabolic pathways in living organisms. Aluminum poisoning can impact blood composition, the musculoskeletal system, kidneys, liver, and respiratory and nervous systems [19]. Therefore, to assess systemic toxicity, body weight was monitored throughout the study (Figure 1). Weekly measurements were taken for all groups, and changes in body weight were expressed as percentages relative to baseline values (Figure 1). Significant weight loss was observed in the AD + Qu_25_ and AD + Qu_50_ groups compared to the healthy control group at 7 days (−11.63 ± 0.83% and −9.9 ± 1.19% vs. 10.61 ± 0.83%, *p* ≤ 0.01, and *p* ≤ 0.05), at 14 days (−9.61 ± 1.46% and −10.24 ± 1.88 % vs.11.73 ± 1.27%, *p* ≤ 0.01, and *p* ≤ 0.01), at 21 days (−13.54 ± 1.12 and −11.85 ± 2.11 vs. 13.01 ± 0.85, *p* ≤ 0.01, and *p* ≤ 0.05) and at 28 days (−10.00 ± 1.28 and −6.46 ± 3.72 vs. 10.04 ± 2.08, *p* ≤ 0.05). Although weight loss was observed in the combined treatment groups compared to the AD model group, the difference was not statistically significant between the groups. The administration of quercetin alone did not result in any significant weight changes compared to the healthy control group.

### 2.2. Quercetin Reduced Serum Iron Levels and Regulated Calcium Homeostasis in Al/D-Gal-Treated Rats

Extensive evidence supports the neurotoxicity of Al, considering it as one of the most hazardous environmental factors related to AD. Aluminum readily crosses the blood–brain barrier (BBB) due to its high affinity for transferrin receptors, leading to disruptions in the homeostasis of essential metals [20]. Considering that quercetin is a powerful antioxidant with metal-chelating properties, our aim was to determine whether quercetin could mitigate metal imbalance and prevent neurotoxicity. Our hypothesis was that quercetin, through its antioxidant effects, metal-chelating ability, and interaction with the Ca^2+^-calmodulin complex, could restore metal homeostasis and reduce neurotoxicity.

Our findings indicate that Al induces neuronal damage by disrupting calcium and iron homeostasis in serum and brain tissue (Table 1). Chronic exposure to Al/D-gal resulted in a significant increase in Al binding to transferrin and serum proteins compared to the healthy control (HC) group (*p* ≤ 0.01). Co-administration of quercetin (Qu) at doses of 25 and 50 mg/kg with Al/D-gal also increased serum Al levels compared to the HC group (both *p* ≤ 0.0001). However, there was no significant difference in aluminum levels between the AD model (Al/D-gal) and the Al/D-gal + Qu groups. However, Qu appears to aid in Al chelation by forming stable complexes that reduce its passage across the BBB. Co-administration of Qu at 25 and 50 mg/kg with Al/D-gal increased serum aluminum retention by 5.29% and 45.12%, respectively. In contrast, aluminum levels in the brain were reduced by 20.83% and 41.67% in the AD + Qu_25_ and AD + Qu_50_ groups, respectively, compared to the AD model. The aluminum concentration in the brain was significantly higher in the AD model compared to the HC group (*p* ≤ 0.001). Interestingly, serum copper levels were increased in all treatment groups compared to the control, while serum iron levels were reduced, especially in the Qu + Al/D-gal combination groups. Increased aluminum accumulation in the brain slightly elevated brain iron levels, while co-administration of quercetin with Al/D-gal reduced brain iron levels in a dose-dependent manner.

Al accumulation in brain tissue also led to a significant increase in Ca^2+^ levels, while co-administration of quercetin restored calcium homeostasis in a dose-dependent manner. Compared to healthy controls, the AD model showed significant calcium dysregulation, which was partially reversed in the AD + Qu_25_ and AD + Qu_50_ groups (*p* ≤ 0.001, *p* ≤ 0.01, and *p* ≤ 0.05, respectively).

**Figure 2:**
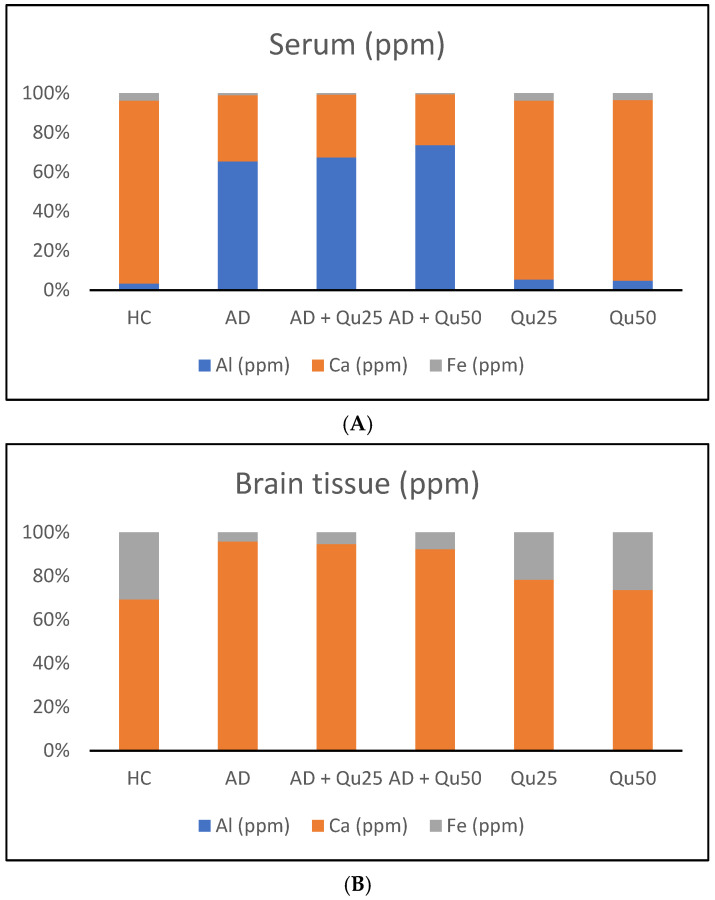

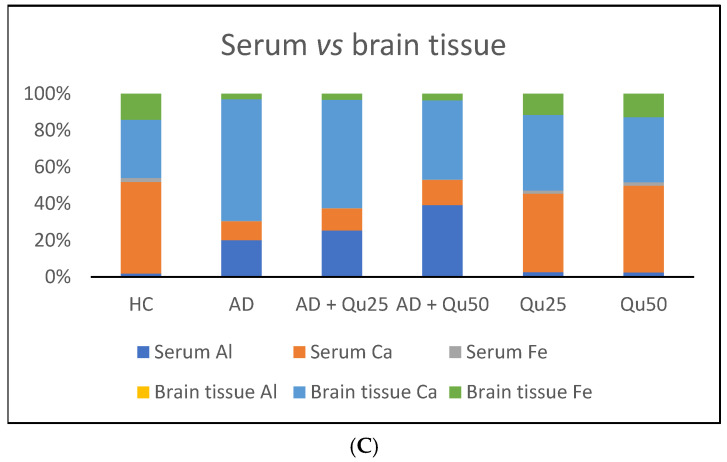
Percentage of aluminum (Al), calcium (Ca), and iron (Fe) in rat serum (**A**), brain tissue (**B**), and serum vs. brain (**C**) after chronic administration of aluminum chloride + D-galactose and quercetin. The rats (n = 3 per group) were injected with aluminum chloride + D-galactose (10 mg/kg + 60 mg/kg) and quercetin at doses of 25 or 50 mg/kg through the intraperitoneal route (*i.p.*) for 28 days. Control rats were injected *i.p.* with saline solution. Data are expressed as a %. Abbreviations: HC—Healthy control group; AD—Alzheimer’s disease rat model; AD + Qu_25_—AD model injected with quercetin *i.p.* at a dose of 25 mg/kg; AD + Qu_50_—AD model injected with quercetin *i.p.* at a dose of 50 mg/kg.

Al toxicity is primarily associated with the disruption of metal homeostasis, such as calcium and iron. Al mimics these metals in biological functions and triggers various biochemical alterations. The observed disturbances in metal homeostasis revealed significant differences in the proportion of individual elements in serum and brain tissue, depending on the treatment and metal accumulation site (Figure 2). In the AD, AD + Qu_25_, and AD + Qu_50_ groups, Al levels in brain tissue were significantly lower than Al values in serum (*p* ≤ 0.001). Similarly, Ca^2+^ levels in brain tissue were significantly higher in the HC, Qu_25_, and Qu_50_ groups compared to the AD, AD + Qu_25_, and AD + Qu_50_ groups (*p* ≤ 0.01). Additionally, Fe values were significantly lower in serum compared to brain tissue across all treated groups (*p* ≤ 0.05).

### 2.3. Quercetin Enhanced Anti-Oxidative Enzymes and Inhibited Oxidative Stress in Al/D-Gal-Treated Rats

**Figure 3:**
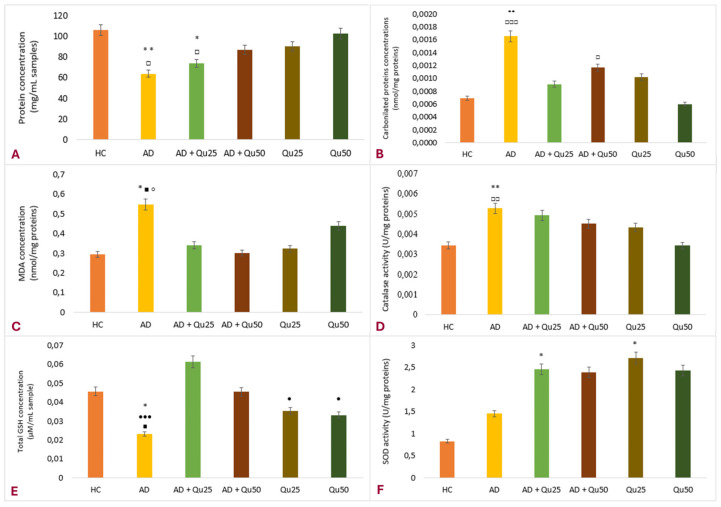
Parameters of oxidative stress and antioxidant protection in rat brain tissue homogenates after chronic administration of aluminum chloride + D-galactose and quercetin. (**A**) Protein concentration; (**B**) Carbonilated proteins concentration; (**C**) MDA concentration; (**D**) catalase activity; (**E**) Total GSH concentration; (**F**) SOD activity. The rats (n = 6 per group) were injected with aluminum chloride + D-galactose (10 mg/kg + 60 mg/kg) and quercetin at doses of 25 or 50 mg/kg through the intraperitoneal route (*i.p.*) for 28 days. Control rats were injected *i.p.* with saline solution. Data are expressed as the mean ± SEM and analyzed using the Kruskal–Wallis nonparametric test. * Significantly different compared to HC (* *p* ≤ 0.05, ** *p* ≤ 0.01). ^□^ Significantly different compared to Qu_50_ (^□^ *p* ≤ 0.05; ^□□^ *p* ≤ 0.01, ^□□□^ *p* ≤ 0.001). ^■^ Statistically different compared to AD + Qu_50_ (^■^ *p* ≤ 0.05). ^○^ Statistically different compared to Qu_25_ (^○^ *p* ≤ 0.05). ^●^ Statistically different compared to AD + Qu_25_ (^●^ *p* ≤ 0.05, ^●●●^ *p* ≤ 0.001). Abbreviations: HC—Healthy control group; AD—Alzheimer’s disease rat model; AD + Qu_25_—AD model injected with quercetin *i.p.* at a dose of 25 mg/kg; AD + Qu_50_—AD model injected with quercetin *i.p.* at a dose of 50 mg/kg; SEM—standard error of the mean.

One of the numerous neurotoxic effects of aluminum is the promotion of oxidative membrane damage. This occurs either through direct interaction with the plasma membrane or indirectly; as a non-redox active metal, Al facilitates the production of OH˙ radicals via the iron-catalyzed Fenton reaction [4,7,8]. Our data demonstrated that the presence of Al in the brain increases ROS levels and reduces the antioxidant capacity of enzymes within brain tissue (Figure 3).

To evaluate the effect of chronic administration of AlCl_3_ + D-gal and Qu on OS parameters (Figure 3), we measured the concentrations of total proteins, carbonylated proteins (PC), and malondialdehyde (MDA) and assessed antioxidant protection by monitoring catalase (CAT) and superoxide dismutase (SOD) activity and the total glutathione (GSH) content in brain tissue homogenates. The results for the total protein concentration in the brain tissue (Figure 3A) showed decreased levels across all treated groups compared to the HC group, but statistically significant differences were found only for AD and AD + Qu_25_ groups compared to HC (*p* ≤ 0.05) and Qu_50_-treated groups (*p* ≤ 0.05). As shown in Figure 3B, significantly higher concentrations of PC were observed in the AD group compared to HC and Qu_50_ -treated groups (*p* ≤ 0.01; *p* ≤ 0.001). The MDA level (Figure 3C), an end product of lipid peroxidation, was increased in the AD group compared to the AD + Q_50_, HC, and Q_25_ groups. CAT enzyme activity (Figure 3D) was also increased in the AD group compared to HC *(p* ≤ 0.01) and Q_50_-treated groups *(p* ≤ 0.01), while the GSH concentration (Figure 3E) was lower compared to the HC, Q_50_, and AD + Qu_25_ groups (*p* ≤ 0.05; *p* ≤ 0.05; and *p* ≤ 0.001, respectively). SOD activity (Figure 3F) was increased in the AD + Qu_25_ and Qu_25_ groups compared to the HC group.

### 2.4. Quercetin Attenuated Al/D-Gal-Induced Neurodegeneration

It has been shown that excessive exposure to Al can lead to the overexpression of amyloid precursor protein (APP) and deposition of Aβ plaques in brain cells. The presence of Al in brain tissue impairs metabolism and ATP production, promotes ROS generation, and induces mitochondrial dysfunction, which in turn leads to a further increase in ROS levels, decreased antioxidant enzyme activity, and neuronal death [8,9,20]. ROS levels and Al toxicity are exacerbated in the presence of metal ions, such as iron, copper, and calcium, which can further increase Aβ aggregation in AD [8,9].

Based on our hypothesis, quercetin could exert a neuroprotective effect by preventing formation and aggregation of Aβ through the following mechanisms: (i) ROS regulation, (ii) scavenging oxygen radicals, (iii) metal chelation, (iv) increase of antioxidant enzymes, and (v) modulation of Ca^2+^ homeostasis.

Neurodegenerative changes were determined by analyzing histological preparations stained using the modified Bielschowski method (silver staining) and immunohistochemical labeling with 4G8, AT8, PHF1, MC1, Iba1, CD68, and HLA-DR markers. Positive signals were observed for the 4G8 and Iba1 markers, while the remaining markers showed either very weak or no signal. As a positive control, brain tissue sections from patients with advanced AD were used, which showed a positive signal for the antibodies used. Sections from the AD group were used as a negative control in the absence of primary antibody labeling (4G8, AT8, PHF1, MC1, Iba1, CD68, and HLA-DR).

**Figure 4:**
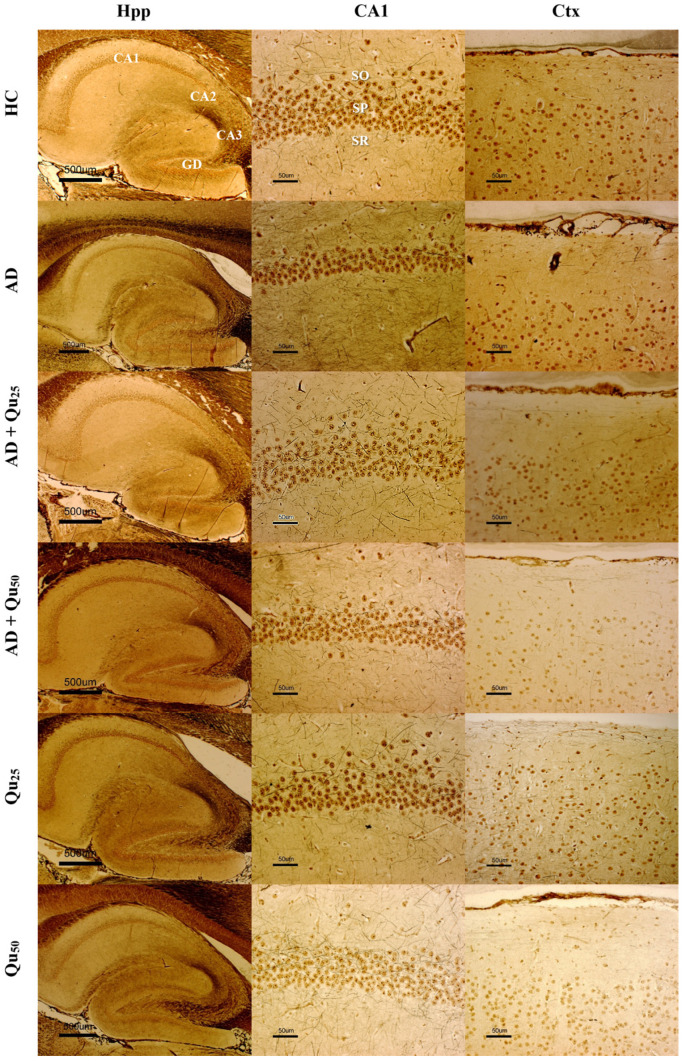
Silver staining of the cortex (Ctx), hippocampus (Hpp), and CA1 area of hippocampal formation in representative cross-sections of rat brains after chronic administration of aluminum chloride + D-galactose and quercetin. The rats (n = 6 per group) were injected with aluminum chloride + D-galactose (10 mg/kg + 60 mg/kg) and quercetin at doses of 25 or 50 mg/kg through the intraperitoneal route (*i.p.*) for 28 days. Control rats were injected *i.p.* with saline solution. The actual magnification is 20× for Ctx and CA1 area (scale bar = 50 µm) and 2× for Hpp (scale bar = 500 µm). Abbreviations: HC—Healthy control group; AD—Alzheimer’s disease rat model; AD + Qu_25_—AD model injected with quercetin *i.p.* at a dose of 25 mg/kg; AD + Qu_50_—AD model injected with quercetin *i.p.* at a dose of 50 mg/kg; Hippocampal formation: GD—*gyrus dentatus* and Ammon’s horn (CA1-CA3, *cornu ammonis*). Layers of the CA1 region: SO—*stratum oriens*, SP—*stratum pyramidale* and SR—*stratum radiatum*.

**Figure 5:**
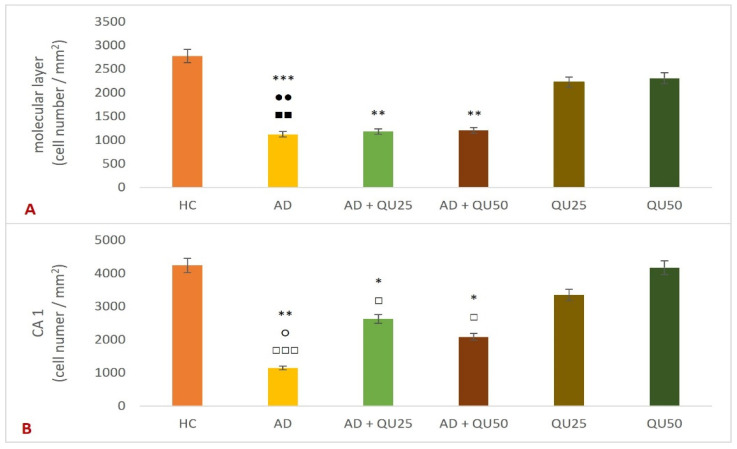
The number of cells in the molecular layer of the cerebral cortex (**A**) and CA1 area of hippocampal formation (**B**) in rat brains after chronic administration of aluminum chloride + D-galactose and quercetin. The rats (n = 6 per group) were injected with aluminum chloride + D-galactose (10 mg/kg + 60 mg/kg) and quercetin at doses of 25 or 50 mg/kg through the intraperitoneal route (*i.p.*) for 28 days. Control rats were injected *i.p.* with saline solution. Data are expressed as the mean ± SEM and analyzed using the Kruskal–Wallis nonparametric test. * Statistical significance compared to HC (* *p* ≤ 0.05, ** *p* ≤ 0.01, *** *p* ≤ 0.001). ^●^ Significantly different compared to AD + Qu_25_ (^●●^ *p* ≤ 0.01). ^■^ Significantly different compared to AD + Qu_50_ (^■■^ *p* ≤ 0.01). ^○^ Significantly different compared to Qu_25_ (^○^ *p* ≤ 0.05). ^□^ Significantly different compared to Qu_50_ (^□^ *p* ≤ 0.05, ^□□□^ *p* ≤ 0.001) Abbreviations: HC—Healthy control group; AD—Alzheimer’s disease rat model; AD + Qu_25_—AD model injected with quercetin *i.p.* at a dose of 25 mg/kg; AD + Qu_50_—AD model injected with quercetin *i.p.* at a dose of 50 mg/kg; SEM—standard error of the mean.

Microscopic analysis of silver-stained preparations (Figure 4) revealed changes in the molecular and outer granular layers of the cerebral cortex and in the hippocampal formation (CA1) in the treated groups compared to the HC group. Quantification of microscopic images showed statistically significant changes in the number of cells in the molecular layer of the cerebral cortex, measured in the reference space between the meninges and the outer granular layer (Figure 5). Statistically significant cell loss in the molecular layer of the cerebral cortex (Figure 5A) was observed in the AD group compared to the HC (*p* = 0.00007), AD + Qu_25_ (*p* = 0.009), and AD + Qu_50_ (*p* = 0.009) groups. Additionally, cell loss remained significant in the AD + Qu_25_ (*p* = 0.007) and AD + Qu_50_ (*p* = 0.009) groups relative to the HC group. A reduced number of cells in the CA1 area of hippocampal formation (Figure 5B) was also evident in the AD group compared to HC (*p* = 0.0012), Qu_25_ (*p* = 0.046), and Qu_50_ (*p* = 0.000012). Furthermore, a reduction in cell count was seen in the AD + Qu_25_ and AD + Qu_50_ groups relative to the HC (*p* = 0.046) and Qu_50_ (*p* = 0.046) groups.

**Figure 6:**
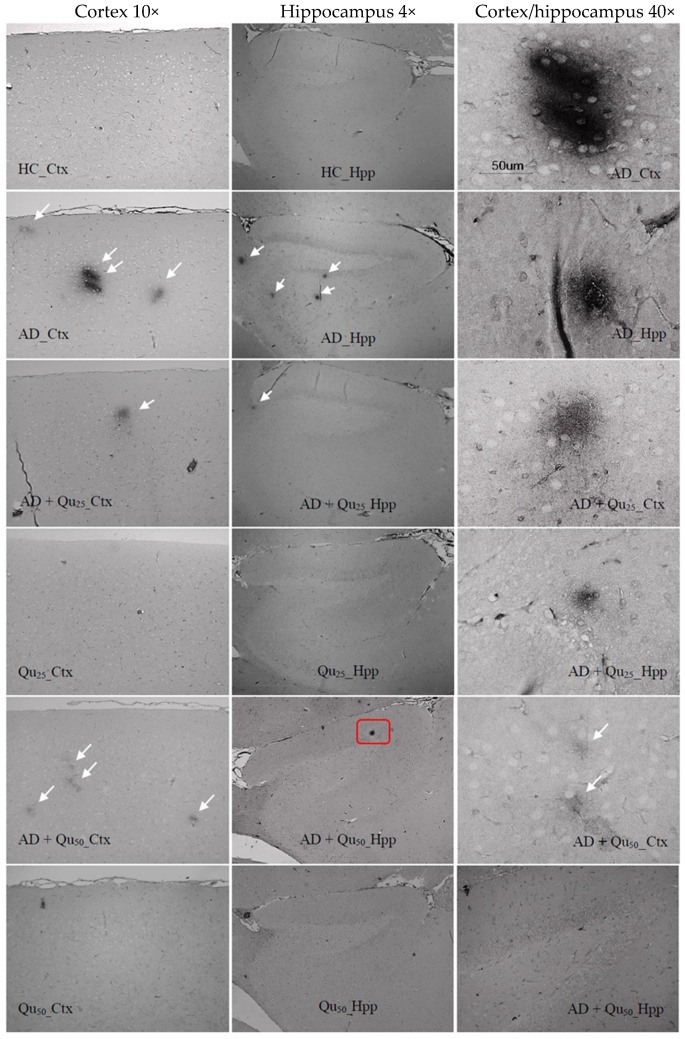
Expression of the 4G8 marker in representative cross-sections of the rat brain cortex and hippocampus, visualized by immunohistochemical staining after chronic administration of aluminum chloride + D-galactose and quercetin. The rats (n = 6 per group) were injected with aluminum chloride + D-galactose (10 mg/kg + 60 mg/kg) and quercetin at doses of 25 or 50 mg/kg through the intraperitoneal route (*i.p.*) for 28 days. Control rats were injected *i.p.* with saline solution. The scale bar for the actual magnification at 40× is 50 µm, with a positive signal indicated by a white arrow. An artifact created during the production of the histological specimen is highlighted by a red rectangle. Abbreviations: HC—Healthy control group; AD—Alzheimer’s disease rat model; AD + Qu_25_—AD model injected with quercetin *i.p.* at a dose of 25 mg/kg; AD + Qu_50_—AD model injected with quercetin *i.p.* at a dose of 50 mg/kg.

**Figure 7:**
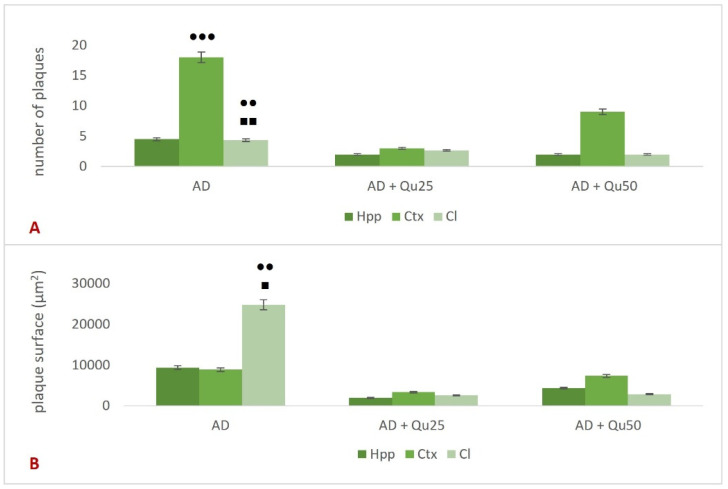
Quantitative analysis of 4G8 marker expression in the brain hippocampal formation, cerebral cortex, and cerebellum of rat brains after chronic administration of aluminum chloride + D-galactose and quercetin. (**A**) Number of plaques; (**B**) Plaque surface. The rats (n = 6 per group) were injected with aluminum chloride + D-galactose (10 mg/kg + 60 mg/kg) and quercetin at doses of 25 or 50 mg/kg through the intraperitoneal route (*i.p.*) for 28 days. Control rats were injected *i.p.* with saline solution. Data are expressed as the mean ± SEM and analyzed using the Kruskal–Wallis nonparametric test. ^●^ Significantly different compared to AD + Qu_25_ (^●●^ *p* ≤ 0.01, ^●●●^ *p* ≤ 0.001). ^■^ Statistically different compared to AD + Qu_50_ (^■^ *p* ≤ 0.05, ^■■^ *p* ≤ 0.01). Abbreviations: HC—Healthy control group; AD—Alzheimer’s disease rat model; AD + Qu_25_—AD model injected with quercetin *i.p.* at a dose of 25 mg/kg; AD + Qu_50_—AD model injected with quercetin *i.p.* at a dose of 50 mg/kg; SEM—standard error of the mean.

The expression of Aβ was analyzed using the 4G8 anti-Aβ antibody. The antibody binds to amino acid residues 17–24 of the Aβ-peptide, recognizing an epitope within amino acids 18–22. The 4G8 anti-Aβ antibody recognizes both misprocessed isoforms and precursor forms of the Aβ peptide. Immunohistochemical staining was performed to analyze the expression of the 4G8 marker (Figure 6) in the cerebral cortex (Ctx) and hippocampal formation (Hpp). The average expression of the marker was higher in the cerebral cortex compared to the hippocampus and was observed in all three groups treated with AlCl_3_ and D-gal (AD, AD + Qu_25_, and AD + Qu_50_). In control samples (HC) and samples treated with quercetin alone (Qu_25_ and Qu_50_), no marker expression was detected. In the hippocampal formation, no statistically significant changes were observed in the number or area of plaques (Figure 7) across the treated groups. However, in the cerebral cortex, a significantly higher number of plaques was observed (Figure 7A) in the AD group compared to the AD + Qu_25_ (*p* = 0.00046). No significant differences were noted in the plaque surface area (Figure 7B). Statistically significant differences were recorded in disease intensity, with the AD group showing higher intensity compared to the AD + Qu_25_ group (*p* = 0.0094).

**Figure 8:**
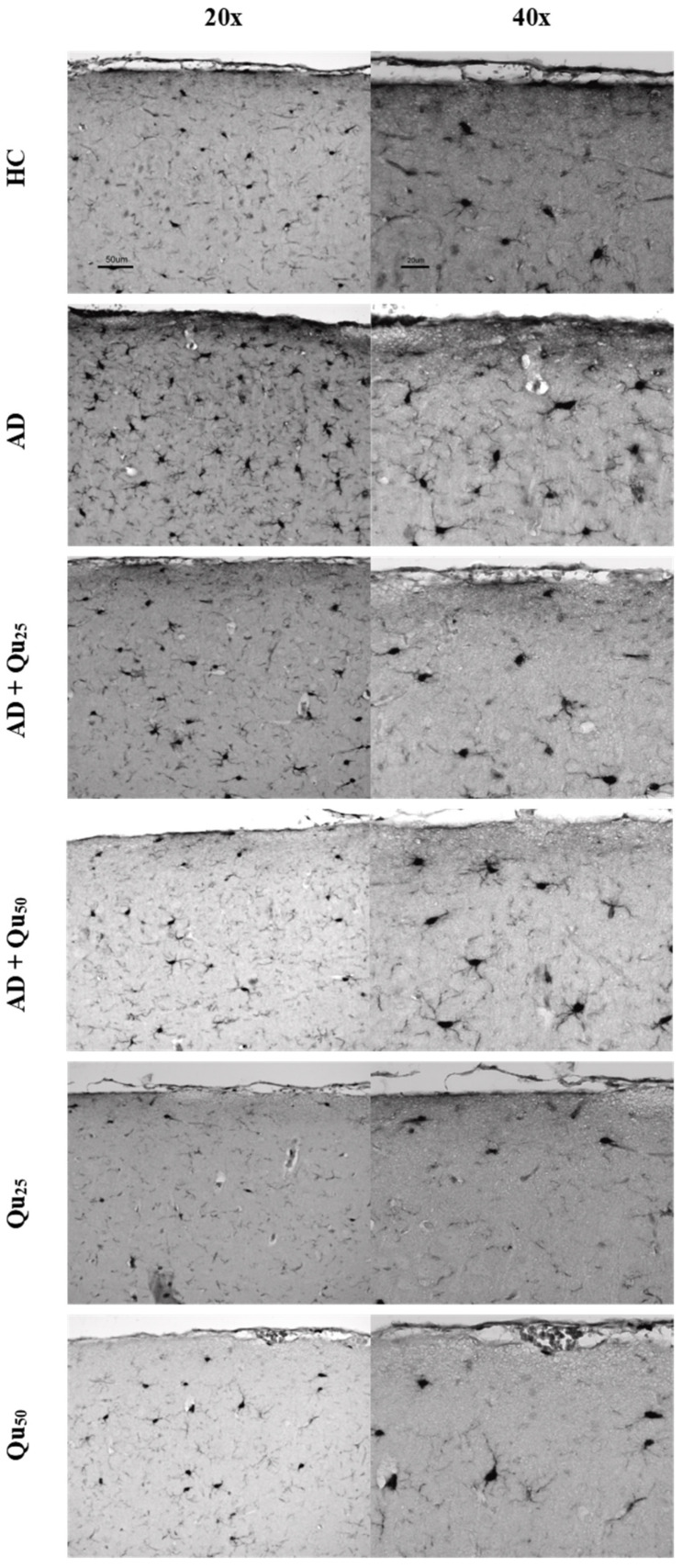
Expression of the Iba1 marker in representative cross-sections of the outer area of the cerebral cortex (Ctx) of the rat brain after chronic administration of aluminum chloride + D-galactose and quercetin. The rats (n = 6 per group) were injected with aluminum chloride + D-galactose (10 mg/kg + 60 mg/kg) and quercetin at a dose of 25 or 50 mg/kg through the intraperitoneal (*i.p.*) route for 28 days. Control rats were injected *i.p.* with saline solution. Actual magnification at 20× (scale bar = 50 µm) and 40× (scale bar = 20 µm). Abbreviations: HC—Healthy control group; AD—Alzheimer’s disease rat model; AD + Qu_25_—AD model injected with quercetin *i.p.* at a dose of 25 mg/kg; AD + Qu_50_—AD model injected with quercetin *i.p.* at a dose of 50 mg/kg.

**Figure 9:**
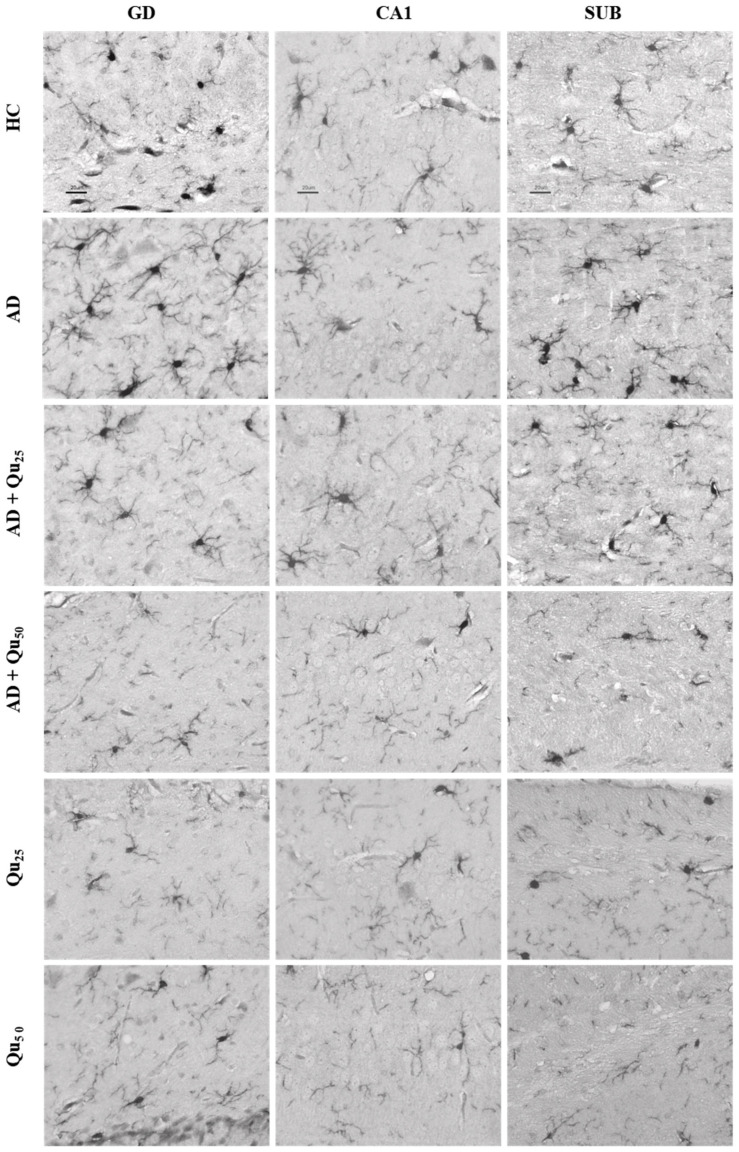
Expression of the Iba1 marker in representative cross-sections of the selected areas of hippocampal formation (GD—gyrus dentatus, CA1—cornu ammonis, SUB—subiculum) of the rat brain after chronic administration of aluminum chloride + D-galactose and quercetin. The rats (n = 6 per group) were injected with aluminum chloride + D-galactose (10 mg/kg + 60 mg/kg) and quercetin at a dose of 25 or 50 mg/kg through the intraperitoneal (*i.p.*) route for 28 days. Control rats were injected *i.p.* with saline solution. Scale bar = 20 µm (40×). Abbreviations: HC—Healthy control group; AD—Alzheimer’s disease rat model; AD + Qu_25_—AD model injected with quercetin *i.p.* at a dose of 25 mg/kg; AD + Qu_50_—AD model injected with quercetin *i.p.* at a dose of 50 mg/kg.

The Iba1 protein plays a crucial role in phagocytosis and membrane folding. This marker identifies microglial cells in both the resting phase and the initial phase of activation, and it clearly highlights the morphology of these cells, particularly the long-branched extensions. The expression of the Iba1 marker is shown in Figure 8 and Figure 9, as well as in Table 2.

A semiquantitative analysis of the microscopic images and quantitative data processing revealed that the expression of the Iba1 marker was significantly increased in the AD group in the cerebral cortex (Table 2) compared to the other treated groups (*p* = 0.0083). In hippocampal formation (Table 2), increased immunoreactivity was observed in the dentate gyrus (GD) of the AD and AD + Qu_25_ groups compared to the HC (*p* = 0.00013) and Qu_50_ (*p* = 0.0083) groups. Additionally, increased immunoreactivity was noted in the CA1 area of the HC, AD, and AD + Qu_25_ groups compared to the AD + Qu_50_, Qu_25_, and Qu_50_ groups (*p* = 0.0012). Finally, in the subiculum (SUB) area, increased immunoreactivity was observed in the HC and AD groups compared to the Qu_25_ and Qu_50_ groups (*p* = 0.00013).

### 2.5. Quercetin Treatment Reverses Dysfunctions of the Cholinergic System and Brain-Derived Neurotrophic Factor (BDNF)

According to several studies [21,22], AD is associated with dysfunctions of the cholinergic system. AChE levels serve as a marker of extensive loss of cholinergic neurons in the brain and were used to evaluate cholinergic dysfunctions. BDNF levels were used to evaluate neuroprotective mechanisms of quercetin, as OS suppresses and damages protective factors, such as BDNF.

**Figure 10:**
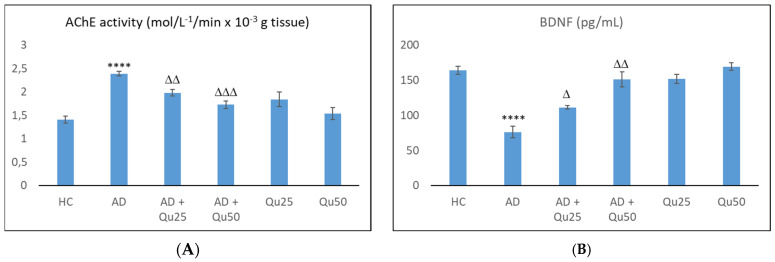
AChE activity (**A**) and BDNF concentration (**B**) in rat brain tissue homogenates after chronic administration of aluminum chloride + D-galactose and quercetin. The rats (n = 3 per group) were injected with aluminum chloride + D-galactose (10 mg/kg + 60 mg/kg) and quercetin at doses of 25 or 50 mg/kg through the intraperitoneal route (*i.p.*) for 28 days. Control rats were injected *i.p.* with saline solution. Data are expressed as the mean ± SEM and analyzed using the Student *t*-test. **** Significantly different compared to the HC group (**** *p* ≤ 0.0001). ^Δ^ Significantly different compared to the AD model (^Δ^ *p* ≤ 0.05; ^ΔΔ^ *p* ≤ 0.01; ^ΔΔΔ^ *p* ≤ 0.01). Abbreviations: HC—Healthy control group; AD—Alzheimer’s disease rat model; AD + Qu_25_—AD model injected with quercetin *i.p.* at a dose of 25 mg/kg; AD + Qu_50_—AD model injected with quercetin *i.p.* at a dose of 50 mg/kg; SEM—standard error of the mean.

Aluminum exposure enhanced the AChE activity, leading to excessive consumption of the acetylcholine and ultimately resulting in cholinergic system deficits (Figure 10). As shown in Figure 10A, the AChE activity was significantly elevated in the Al/D-gal treated group compared to the HC group (*p* ≤ 0.0001). Treatment with quercetin significantly reduced AChE levels in both AD + Qu_25_ (*p* ≤ 0.01) and AD + Qu_50_ (*p* ≤ 0.001) groups. There was no significant difference in AChE levels between the HC and quercetin-treated groups.

In contrast to AChE, BDNF levels were lowest in the AD group compared to the HC group (*p* ≤ 0.0001), while quercetin treatment increased BDNF levels in all groups, demonstrating its protective effect. Co-administration of quercetin with Al/D-gal resulted in a 46.26% (*p* ≤ 0.05) and 98.30% (*p* ≤ 0.01) increase in BDNF levels at doses of 25 and 50 mg/kg, respectively.

### 2.6. Quercetin Reduces Al/D-Gal-Induced Neuroinflammation

Al also exerts pro-oxidative, excitotoxic, immunogenic, proinflammatory, and mutagenic effects. In AD, the neuroinflammatory response mediated by microglial cells plays a key role in the pathogenesis of the disease. Prolonged activation of microglia results in the release of pro-inflammatory cytokines, such as interleukin (IL)-1β, IL-6, IL-12, interferon (IFN)-γ, and tumor necrosis alpha (TNF-α), triggering a pro-inflammatory cascade that ultimately leads to neural damage or loss.

The results of the pro-inflammatory and anti-inflammatory cytokine analysis are shown in Table 3. Al/D-gal treatment significantly increased the levels of IL-1α, IL-1β, IL-2, IL-4, IL-6, IL-10, IL-12, IL-13, IFN-γ, and TNF-α and granulocyte-macrophage colony stimulating factor (GM-CSF) compared to the HC group (*p* ≤ 0.01). In the AD + Qu_25_ group, IL-4 and chemokine regulated upon activation, normal T-cell expression, and secretion (RANTES) levels were also elevated compared to the HC group (*p* ≤ 0.05 for both). Co-administration of quercetin with Al/D-gal reduced the levels of inflammatory cytokines in a dose-dependent manner. Quercetin at a dose of 50 mg/kg significantly decreased the concentrations of IL-1β, IL-2, IL-6, IL-10, and TNF-α compared to the Al/D-gal group (*p* ≤ 0.05).

Neuroinflammation was further confirmed through the relative brain index (RBI), which increased by approximately 20% in the AD and AD + Qu_25_ groups compared to the HC group, while in the AD + Qu_50_ group, the increase was only 7% (see Supplementary Data).

### 2.7. Results of Neurological Screening

Motor and sensory perception were assessed before and after chronic administration of AlCl_3_ + D-gal and Qu using the standard neurological screening (NS) protocol. The NS was performed to determine whether any observed changes were part of the existing pathology or induced by the treatment. Table 4 shows the results recorded 24 h prior to and after the treatment. The analysis of the neurological examination did not reveal any significant deviations in motor or sensory perception before or after chronic administration of AlCl_3_ + D-gal and Qu.

## 3. Discussion

AD is a progressive neurodegenerative disease and the most common type of dementia that affects memory, cognition, and behavior. It is estimated that over 55 million people have AD, which usually results in death within 3–9 years after diagnosis [23]. Despite extensive research efforts, the exact underlying cause of AD remains unknown. However, several risk factors, such as aging, oxidative stress, neuroinflammation, and chronic exposure to environmental metal toxicants, particularly Al, cadmium, and lead, have been associated with the onset and progression of the disease [24]. Aluminum is a metal that is toxic to patients with end-stage renal disease who are on long-term hemodialysis, where aluminum causes a number of clinical disorders, including mental and language disorders, behavioral disorders, cognitive decline, and movement disorders [12]. It has been observed that if the dialysis water is not double demineralized and distilled, excess aluminum in the dialysate rapidly causes small, flake-like amyloid plaques [13]. It is puzzling that despite these clinical symptoms, focal neurological signs and mild lesions are rarely observed in brain pathology. Among these, Al has emerged as a particularly potent neurotoxin [25,26]. Due to its ability to easily cross the BBB and its high affinity for transferrin receptors, Al can accumulate in various brain regions and induce free radical production, leading to brain cell damage, especially in areas involved in learning and memory [9,27]. ROS-induced neurotoxicity is the key pathological event in AD. As there is still no satisfactory therapy for AD, powerful natural antioxidants, such as quercetin, are being considered as a potential option for preventing the onset and progression of AD.

The combined administration of AlCl_3_ and D-gal is a well-established model for inducing neurotoxicity in animals. This model induces strong OS and inflammation by increasing the pro-oxidative iron activity and suppressing the action of antioxidant enzymes in the brain [7,8,9,27]. In addition to its pro-oxidant and pro-inflammatory properties, Al also affects the homeostasis of calcium, phosphorus, copper and iron, and has an adverse effect on essential elements [8,27]. The level of ROS and the kinetics of Al toxicity are exacerbated by the activation of Fe^2+^ and Fe^3+^ ions. Al also induces neuronal injury by interfering with calcium homeostasis; it delays the closure of voltage-dependent calcium channels and inhibits calmodulin (CaM)-dependent Ca^2+^/Mg^2+^-ATPase, thereby impairing the protective mechanisms against excitotoxicity [27,28,29].

Our data are consistent with previous findings, showing that Al/D-gal administration results in reduced body weight, elevated lipid peroxidation, increased PC levels, and higher AChE activity in the brain (Figure 1, Figure 3 and Figure 10). These changes contribute to the increased accumulation of free radicals and OS, ultimately leading to decreased BDNF levels and increased neurotoxicity (Figure 4, Figure 5, Figure 6, Figure 7, Figure 8, Figure 9 and Figure 10). Al/D-gal-induced ROS (Figure 3C) cause cellular damage by oxidizing amino acid residues, forming PCs (Figure 3B), and depleting the most important cellular antioxidant GSH (Figure 3E).

According to Kim et al. [30], aluminum disrupts iron homeostasis in the brain via multiple mechanisms, including transferrin receptors, non-transferrin iron transporters, and ferritin. Aluminum increases the uptake of both non-transferrin-bound and transferrin-bound iron while decreasing ferritin-bound iron. It can also bind to at least one of the two specific iron-binding sites of serum transferrin and serum albumin. Free iron, in turn, catalyzes ROS reactions (Figure 3), producing hydroxyl radicals and lipid peroxides, depleting GSH, impairing antioxidant enzyme activity, and triggering cell death pathways, such as ferroptosis. These processes contribute to the observed decline in protein levels in the brain (Figure 3A). Free intracellular iron promotes peroxidation of membrane lipids and increases protein carbonylation, resulting in membrane damage (Figure 3B,C), apoptosis, and necrosis.

Interestingly, chronic Al/D-gal treatment caused a significant increase in CAT activity (Figure 3D). Of note, CAT is a primary defense enzyme against oxidative injury [31] that works synergistically with SOD to neutralize ROS and prevent tissue damage. SOD facilitates the conversion of superoxide radicals to hydrogen peroxide, while CAT converts hydrogen peroxide into water and molecular oxygen (Figure 3F).

Quercetin treatment significantly reduced Al/D-gal-induced ROS production (Figure 3), indicating its potential to modulate downstream targets of ROS implicated in neurodegeneration (Figure 4, Figure 5, Figure 6, Figure 7, Figure 8, Figure 9 and Figure 10). Quercetin is a potent inhibitor of Fe^2+^-induced lipid peroxidation in rat serum and brain homogenates (Table 1, Figure 2), and it markedly reduced ROS production in the brains of Al/D-gal-treated rats. This reduction in ROS levels subsequently alleviated OS and restored several biochemical markers, including the PC content, GSH levels, and the activities of CAT, SOD and AChE, toward normal levels (Figure 3).

Previous studies have shown that quercetin exhibits several antioxidant properties, including radical scavenging activity (DPPH, OH, and NO), Fe^2+^ chelation, and inhibition of Fe^2+^-induced lipid peroxidation. Furthermore, it upregulates the expression of antioxidant enzymes by modulating the nuclear factor erythroid 2-related factor 2 (Nrf2) signaling pathway, likely contributing to the restoration of cellular redox balance disrupted by Al/D-gal treatment in the brain [10,14].

Al/D-gal acts as a strong cholinotoxin that mimics age-related cholinergic dysfunction in rats. It significantly enhances the AChE activity (Figure 10), leading to accelerated breakdown of ACh in the brain, which is associated with cognitive deficits, and impaired learning and short-term memory [9,27]. Interestingly, administration of 25 or 50 mg/kg quercetin to Al/D-gal-treated animals significantly suppressed AChE activity (*p* ≤ 0.01, *p* ≤ 0.001) (Figure 10). These data coincide with previous findings [32,33,34] demonstrating quercetin’s AChE inhibitory and Fe^2+^- chelating activities. According to Abdalla et al. [34], quercetin inhibits AChE via hydrophobic interactions and strong hydrogen bonding with the enzyme, thereby reducing ACh hydrolysis and increasing ACh levels in the synaptic cleft. The inhibition of cholinesterases, together with prominent antioxidative properties, such as strong radical scavenging ability, may underline neuroprotective effects of quercetin and other flavonoids. These mechanisms suggest that flavonoids, including quercetin, could be promising agents in managing OS-induced neurodegeneration. Furthermore, by inhibiting AChE activity, quercetin may help prevent Aβ aggregation, as increased AChE activity is known to accelerate the formation of Aβ (Figure 6).

Excessive exposure to Al, together with OS and inflammation, lead to the APP overexpression and increased deposition of Aβ plaques in brain tissue (Figure 6 and Figure 7), while neurofibrillary tangles pathology was not detected. Al/D-gal also disrupted intracellular metal homeostasis (e.g., Ca, Fe, Cu, and other essential metals), promoting inflammation and the production of pro-inflammatory cytokines (Table 3) and ultimately lowering BDNF, which is crucial for neuronal survival and differentiation [21,35,36]. The inflammatory response activated microglia and astrocytes, resulting in further production of ROS, DNA fragmentation, neuroinflammation, chronic OS, and ultimately cell death (Figure 5, Table 2 and Table 3). An increase in ROS production can trigger Aβ aggregation, a hallmark of AD (Figure 6). Aβ plaques are thought to result, at least in part, from insufficient clearance by microglia. Notably, microglia cells have a dual role in AD; while they mediate Aβ-induced neuroinflammation and contribute to cognitive decline, they are also involved in Aβ clearance by phagocytosis [37].

HLA-DR, Iba1, and CD68 are widely used markers for characterizing different stages of microglial activation. According to Hendrickx et al. [38], HLA-DR and CD68 markers indicate immune activation and the response to tissue damage, while Iba1 is more suitable for structural studies in the absence of pathology. In our study, we detected only Iba1 in microglia located in areas with diffuse plaque formation (Figure 8 and Figure 9, Table 2). These results suggest that Iba1 may serve as an early activation marker of microglia, while HLA-DR and CD68 appear more strongly associated with dense-core senile plaques. These findings are consistent with the neurological screening data (Table 4).

Dysregulation of cerebral metal ions, such as iron, copper, and calcium, has been recognized as a key early event in the aggregation of Aβ (Figure 5). Ferric iron can interact with Aβ peptides and promote the aggregation of both Aβ40 and Aβ42. Calcium ions can also promote the formation of Aβ peptide and accelerate the aggregation process. Furthermore, Aβ oligomers can incorporate into the cell membrane, forming amyloid channels that facilitate the influx of calcium. This, in turn, leads to enhanced tau phosphorylation, reduction of neurotrophic factors, increased OS, and ultimately, neuronal death. The gradual accumulation of Cu^2+^ and Fe^2+^ in amyloid plaques is closely linked to OS [39]. Also, metal ion levels in AD are often significantly higher compared to healthy controls [39].

Elements play various roles in biological systems, from participating in metabolic reactions to modulating OS. For example, Fe, a redox-reactive metal, plays a critical role in maintaining normal brain function. It contributes to NO metabolism, neurotransmitter synthesis, and myelination, among others [39]. However, changes in serum levels of various elements are associated with the etiology and pathophysiology of many diseases. As mentioned previously, transition metals, such as iron and copper, can drive the formation of hydroxyl radicals via the Fenton reaction, leading to damage of essential biological macromolecules and promoting OS. On the contrary, selenium and manganese are important components of the antioxidant enzyme system [39,40,41].

As shown in our results (Table 2), chronic exposure of animals to Al/D-gal leads to significantly higher binding of Al to transferrin and serum proteins compared to the healthy control group (*p* ≤ 0.01). Co-administration of quercetin at doses of 25 and 50 mg/kg with Al/D-gal increased serum aluminum retention by 5.29% and 45.12%, respectively, compared to the AD model. This suggests that quercetin may help chelate aluminum by forming stable, polar complexes, thereby reducing its passage across the BBB [14,42]. Quercetin presents the three complexing sites (α-hydroxy-carbonyl, β-hydroxy-carbonyl, and catechol) in competition towards the Al(III) fixation [42].When complexed with metal ions, quercetin also demonstrates potent antioxidant activity [14,42].

In the brain, co-administration of quercetin (25 and 50 mg/kg) with Al/D-gal reduced aluminum accumulation by 20.83% and 41.67%, respectively, compared to the AD model. Furthermore, accumulation of Al in brain tissue was associated with a significant increase in Ca levels. Quercetin administration reversed this effect in a dose-dependent manner, restoring Ca ion homeostasis (Table 2, Figure 2). Disruption of calcium homeostasis is recognized as an early and critical event in AlCl_3_-induced neurotoxicity and neurodegeneration [43]. Studies have shown that AlCl_3_ inhibits voltage gated calcium channels (VGCCs), promoting increased intracellular calcium influx and contributing to neuronal excitotoxicity [8,39,43]. Calcium plays a central role in neurotransmitter release, gene expression, and apoptosis [9,11,12]. Disturbed Ca homeostasis within the endoplasmic reticulum and mitochondria leads to mitochondrial dysfunction and neurodegeneration, exacerbating damage to the mitochondrial energy system. Although calcium is essential for mitochondrial ATP production, elevated mitochondrial calcium levels can trigger the opening of the mitochondrial permeability transition pore (mPTP), leading to apoptosis. In addition, excessive Ca^2+^ can overactivate glutamate receptors, such as N-methyl-D-aspartate (NMDA) receptors, causing excitotoxicity and neuronal damage through OS and calcium overload [12,13]. Taken together, calcium dysregulation contributes to AD progression through multiple mechanisms: (i) the disturbance of calcium homeostasis may cause damage to neuronal structures, leading to cell necrosis and dysfunction; (ii) increased intracellular calcium promotes Aβ deposition; (iii) calcium overload causes abnormal tau phosphorylation and inhibits its binding to microtubules, resulting in neurofibrillary tangle formation; and (iv) disruption of calcium homeostasis leads to abnormal synaptic plasticity, contributing to cognitive impairment in AD patients [44].

Our data also indicate that ROS levels and the kinetics of Al toxicity are exacerbated in the presence of cerebral metal ions, such as iron, copper, and calcium, which may promote Aβ aggregation (Figure 6 and Figure 7). Increased OS, Aβ oligomer formation and aggregation, and the development of plaques in the cortex and hippocampus appear to result from Al accumulation and disrupted homeostasis of calcium, Fe, and other essential elements [39,40,41,42,43,44]. The lack of tau pathology observed in our Al/D-gal-induced AD model is most likely related to the genetic background of the animals rather than to the low aluminum concentration used. Additional contributing factors may include the specific form of aluminum administered (e.g., chloride, lactate, maltolate), the route of administration, duration of exposure, and the age of the animals. These variables are known to influence the development of tau-related pathology in experimental models [4,9,10,11].

Quercetin treatment was associated with the reduced Aβ accumulation and attenuated astroglial and microglial reactivity in the cerebral cortex and hippocampal regions (CA1, GD, SUB) of rat brains (Figure 8 and Figure 9, Table 4). A high dose of quercetin also significantly reduced serum and brain levels of Fe, likely by binding non-heme Fe, chelating free Fe ions, and increasing transferrin levels [45,46]. According to our findings (Figure 4), impaired metal homeostasis was evident in both the brain and serum, with the proportion of individual elements (%) varying by treatment and the site of metal accumulation. By regulating Ca homeostasis, quercetin may interfere with the formation of neurotoxic oligomeric Aβ species, destabilize fibrils, and reduce Aβ-induced neurotoxicity [45,46]. Its scavenging capacity is enhanced when complexed with copper, iron, and calcium, showing better antioxidant activity compared to quercetin alone, as demonstrated by DPPH free radical scavenging assays. The enhanced antioxidant activity of quercetin complexes supports its potential application in medicine [44], as confirmed by our results of OS markers (Figure 3). Both our previous research and this study demonstrated that quercetin exerts neuroprotective effects and inhibits the formation and aggregation of Aβ through several mechanisms: (i) regulation of ROS, (ii) scavenging oxygen radicals, (iii) chelation of metal ions, (iv) enhancement of antioxidant enzyme activity, and (v) modulation of Ca^2+^ homeostasis. The molecular structure of quercetin plays a crucial role in its ability to inhibit Aβ aggregation, particularly due to the presence of two aromatic rings connected by linkers of two to six atoms. Aromatic rings with at least three hydroxyl groups are essential for inhibiting fibril formation via hydrophobic interactions between the aromatic rings and the β-sheet structures, forming hydrogen bonds. The high number of hydroxyl groups in quercetin’s structure enhances its anti-amyloidogenic potential, with more hydroxyl groups correlating with better efficacy [14,15,16,17,18]. Phenolic hydroxyls increase electron density in the aromatic rings, thus strengthening the binding of quercetin to aromatic amino acids within β-sheet structures. Quercetin binds to β-amyloid oligomers at early stages of aggregation, promoting the formation of modified oligomers and impeding β-sheet formation, potentially delaying the onset of AD. In addition, quercetin has demonstrated greater inhibitory activity against Aβ aggregation compared to other flavonoids, such as kaempferol, morin, and datisketin [14,15,16,17,18,45,46]. This enhanced activity is attributed to the autoxidation of its catechol structure to o-quinone on the B ring, which then forms the o-quinone-Aβ42 adduct by targeting Lys residues at positions 16 and 28 of Aβ42. Another important mechanism by which quercetin inhibits Aβ formation involves inhibition of the nuclear factor kappa-light-chain-enhancer of the activated B cells (NF-κB) pathway. NF-κB regulates Aβ production by controlling β-cleavage of the APP. By inhibiting NF-κB, quercetin reduces the activity of the beta-secretase-1 (BACE-1) enzyme activity via hydrogen bond interactions, with the OH group at the C-3 position being especially critical. Furthermore, quercetin impairs APP maturation, thus altering Aβ synthesis and aggregation [47]. Taken together, our data suggest that quercetin may be considered as a promising drug candidate for AD, as it effectively inhibits Aβ formation and aggregation in the hippocampal formation, cerebral cortex and cerebellum (Figure 9).

Given that Al and other heavy metals can reach the neuronal environment, they are capable to disturb redox balance and interfere with the cellular microenvironment and neurotrophic signaling. Following Al administration, we observed a significant reduction in BDNF compared to healthy controls (Figure 10). BDNF plays an important role in regulating the survival and differentiation of a specific neuronal populations. It is abundantly expressed in the hippocampus and cortex, two brain regions essential for learning and memory [48]. BDNF also has trophic effects on serotonergic neurons in the CNS. Decreased expression of BDNF has been associated with impaired neuronal circuitry, playing an important role in the development of neurodegenerative diseases [14,21,36]. Consistent with previous studies, our findings confirm that Al intoxication reduces BDNF levels [14,21,36,48]. In addition, Al impairs activity of various enzymes involved in neurotransmitter biosynthesis and may affect serotonergic neurotransmission in the hippocampus [36]. Al can reduce serotonin levels either directly by inhibiting its synthesis or indirectly by reducing BDNF levels [36].

Although low BDNF levels in AD are widely recognized as a marker of neurodegeneration and disease progression, quercetin treatment restored BDNF levels in Al/D-gal induced AD rat model (Figure 10). Treatment with quercetin increased BDNF levels in all groups, showing its protective effect on BDNF expression. Co-administration of quercetin at doses of 25 or 50 mg/kg with Al/-D-gal increased BDNF levels by 46.26% (*p* ≤ 0.05) and 98.30% (*p* ≤ 0.01), respectively. These findings demonstrate that Qu can dose-dependently reverse the adverse effects of AlCl_3_, with BDNF levels in the AD + Qu_25_ and AD + Qu_50_ groups differing from the healthy control group by 32.07% and 7.89%, respectively. Although numerous studies have confirmed the positive effects of quercetin on BDNF levels in various pathological conditions, our results showed no significant change in BDNF expression in healthy rats treated with quercetin compared to the HC group. Previous research has shown that administration of quercetin-3-O-glucuronide, the main metabolite of quercetin, increases hippocampal neurogenesis in adult mice [49]. Furthermore, daily administration of 14–16 mg of quercetin to rats for one month promoted neural stem cell proliferation and differentiation by increasing the BDNF expression [50]. Quercetin has also been shown to prevent BDNF downregulation in the hippocampus of rats exposed to hypobaric hypoxia [51].

As mentioned, AD is characterized by chronic inflammation of microglia, astrocytes, and peripheral immune cells due to the accumulation of APs and NFTs in the brain [14,22,35]. Misfolded and aggregated proteins bind to pattern recognition receptors on microglia and astrocytes, triggering an immune response and releasing numerous inflammatory cytokines and chemokines [35,36]. Aβ and APP induce the release of these signaling molecules from microglia, astrocytes and neurons. Proinflammatory cytokines, such as IL-1β, IL-6, IL-10, and TNF-α, were measured in both serum and brain tissue [35]. These proinflammatory cytokines play a key role in the pathogenesis of AD [14,22,35,36] and serve as biomarkers of the disease state and therapeutic response. Overexpression of IL-1β and IL-6 in microglia cells is thought to result from Aβ accumulation, while IL-6, despite being a proinflammatory cytokine, may also exert neuroprotective effects under specific conditions. IL-6, together with IL-10, the main anti-inflammatory cytokine associated with the onset of AD, can help resolve the inflammatory cascade and support brain integrity and neurogenesis. However, prolonged production of IL-10 may suppress T helper cell responses. Accordingly, IL-10 plays a crucial role in modulating neuroinflammation and is considered an important biomarker for both the diagnosis and progression of Alzheimer’s disease.

Granulocyte-macrophage colony-stimulating factor (GM-CSF) is a pleiotropic cytokine that is upregulated in various neurological disorders, including Alzheimer’s disease, vascular dementia, and multiple sclerosis. This cytokine stimulates the proliferation of microglial cells and contributes to CNS inflammation; its levels correlate with disease severity in inflammatory conditions. However, the exact mechanisms by which GM-CSF contributes to the neuroinflammatory response remain unclear, as it does not appear to induce production of typical pro-inflammatory mediators, such as NO and TNF-α. Some studies suggest that GM-CSF may trigger programmed cell death in the brain tissue of dementia patients. Conversely, GM-CSF also participates in T cell-mediated immunomodulation of microglia, promotes Aβ clearance, helps maintain synaptic integrity, and supports neurogenesis. Therefore, neurodegenerative changes and the accelerated process of neuroinflammation, aging, and cognitive decline in AD may be attenuated by GM-CSF treatment. The overall effects of GM-CSF vary depending on the disease state and the immune environment [14,22,35,36].

Increased immunoreactivity of microglial cells was confirmed in our AD model (Figure 8 and Figure 9) by the expression of the Iba1 marker. According to our findings, rats exposed to Al/D-gal showed increased levels of IL-1α, IL-1β, IL-2, IL-4, IL-6, IL-10, IL-12, IL-13, IFN-γ, TNF-α, and GM-CSF cytokines (Table 3), consistent with previous reports [14,22,35,36]. As previously mentioned, quercetin reduced Aβ aggregation and microglial immunoreactivity (Figure 8 and Figure 9). Co-administration of quercetin at a dose of 50 mg/kg significantly reduced the levels of IL-1β, IL-2, IL-6, IL-10, and TNF-α compared to the AD model group (Table 3). These results highlight the considerable potential of quercetin in reducing neuroinflammation and protecting against Al/D-gal-induced neurotoxicity. In addition, quercetin attenuated the Al/D-gal-induced increase of TNF-α, which is shown to exacerbate glutamate-mediated cytotoxicity by inhibiting glutamate uptake in astrocytes and increasing the surface expression of Ca^2+^-permeable AMPA and NMDA receptors, while simultaneously decreasing inhibitory GABA_A_ receptors on neurons [14,52]. This multifaceted action of quercetin, including antioxidant, anti-inflammatory, and anti-amyloid properties, and the ability to regulate both AChE and BDNF, underscores its potential as a therapeutic target in the treatment of AD. Similar neuroprotective and anti-inflammatory properties are also shown by other natural components, including isocoumarins [5,18,22,27,32,36,53,54].

Although the rat model offers several advantages, such as its size, the presence of all six tau isoforms similar to humans, and relatively easy breeding and maintenance, it also has several limitations: (i) the specific mechanisms of neuroprotection in rats are not fully understood, and effects can vary depending on the strain or model used; (ii) inherent differences in brain size, structure, and function between rat and humans, which may influence drug responses; (iii) genetic variability and differences in drug and disease response; (iv) challenges in translating findings from rats to humans; (v) relatively short life span of rats, requiring longer exposure periods to study long-term effects; (vi) certain conditions are difficult to replicate accurately in rats due to the complexity of human diseases; (vii) differences in the bioavailability and metabolism of certain substances; and (viii) the rate of regeneration and healing is different between rats and humans.

Future research will focus on investigating the long-term effects of quercetin on AD, as well as the potential connection between quercetin’s efficacy and gut microbiota composition. Considering the critical role of the intestinal microbiota in maintaining overall health and regulating vital homeostatic processes in CNS, it is essential to investigate the bidirectional communication system known as the “gut–brain axis”. Given that quercetin is found in numerous fruits and vegetables, changes in the gut microbiota induced by quercetin may affect neural, humoral and inflammatory pathways, with outcomes depending on both host and environmental factors.

## 4. Materials and Methods

### 4.1. Research Animal Study Design

The study was conducted on male Y59 rats, aged 3 months and weighing 270–300 g, obtained from the Department of Animal Physiology, Faculty of Science, University of Zagreb. The animals were kept under standard conditions (12-h light/dark cycle, temperature 24 °C ± 2 °C, and controlled humidity at 60%). The rats were fed a standard laboratory diet (Standard Diet GLP, 4RF21, Mucedola, Settimo Milanese, Milan, Italy), with food and water available ad libitum. The experiments were approved by the Ethics Committee of the Faculty of Science, University of Zagreb (approval number: 251-58-10617-16-25, date: 5 October 2016). The research was conducted in accordance with the ethical and legal principles valid in the Republic of Croatia (Law on Animal Welfare, NN 102/2017; Law on Amendments to the Law on Animal Welfare, NN 37/13; Regulation on the Protection of Animals Used for Scientific Purposes, NN 55/13 and Guide for the Care and Use of Laboratory Animals, DHHS (NIH) Publ. No. 86-23, National Research Council.

A total of 60 rats were used in the study. The animals were randomly divided into 6 groups (each group containing 10 rats) and housed in stainless steel cages (5 rats per cage) under the same controlled conditions. Physiological saline was used as the vehicle for quercetin and aluminum. Both solutions were freshly prepared and administered intraperitoneally (*i.p.*) in a volume of 0.5 mL. The rats were treated for 28 days as follows (see Table 5):

1. Healthy control group (HC): control rats injected intraperitoneally (*i.p.*) with saline solution.
2. AD rat model (AD): rats injected *i.p.* with aluminum chloride at a dose of 10 mg/kg and D-galactose at a dose of 60 mg/kg (Al/D-gal).
3. Aluminum + quercetin-treated group (AD + Qu_25_): AD model rats injected *i.p.* with quercetin at a dose of 25 mg/kg.
4. Aluminum + quercetin-treated group (AD + Qu_50_): AD model rats injected *i.p.* with quercetin at a dose of 50 mg/kg.
5. Quercetin-treated group (Q_25_): Rats injected *i.p.* with quercetin at a dose of 25 mg/kg.
6. Quercetin-treated group (Q_50_): Rats injected *i.p.* with quercetin at a dose of 50 mg/kg.

Twenty-four hours after the last injection, the rats were anesthetized using a mixture of ketamine (Narketan 10, Vetoquinol AG, Belp Bern, Switzerland) at a dose of 75 mg/kg and xylazine (Xylapan Vetoquinol Biowet Sp., Gorzów, Poland) at a dose of 10 mg/kg. Following blood sampling, the rats were sacrificed by exsanguination, and the organs and samples of interest were collected. Serum was separated from the blood by centrifugation at 3000× *g* for 10 min at 4 °C and stored in 500 μL aliquots at −80 °C until further use. Brain tissue and blood samples for inductively coupled plasma-mass spectrometry (ICP-MS) were stored in polypropylene tubes with a stoppers at −20 °C until analysis. For spectrophotometric analysis, serum and brain tissue samples were stored in 2 mL polypropylene tubes at −80 °C.

### 4.2. Test Components

Aluminum chloride hexahydrate (AlCl_3_·6H_2_O, Mr = 241.45 g/mol, purity > 95%) was obtained from Gram-mol d.o.o., Zagreb, Croatia. D(+)-galactose for microbiology (C_6_H_12_O_6_, Mr = 180.16 g/mol, purity > 97%) was obtained from Merck, Darmstadt, Germany. Quercetin dihydrate (C_15_H_10_O_7_·2H_2_O, Mr = 338.27 g/mol, purity > 98%) was obtained from Sigma-Aldrich Corporation, Germany. Sodium Chloride 0.9% Infusion Solution was manufactured by B. Braun Adria d. o. o., Croatia.

### 4.3. Animal Weight Changes and Relative Brain Weight

The Al/D-gal-induced toxicity at the organism level was assessed by monitoring the body weight of each animal. The animals were weighed using a digital scale (Kern KB 2000-2N, precision: 0.01 g, maximum capacity: 2000 g) at the following time points: (i) at the beginning of the experiment, (ii) every seven days during the treatment period, and (iii) on the day of sacrifice. Weight loss was calculated for each animal using the following formula:

(1) $$Percentage\;change\;in\;weight=\frac{\left(Final\;weight-Initial\;weight\right)\times 100}{Final\;weight}$$

After sacrifice, the brain was isolated and weighed using a digital scale (Analytical balance ABS 220–4, Kern & Sohn GmbH, Balingen, Germany). The recorded values of brain mass and body mass were used to calculate the relative brain weight using the following formula:

(2) $$Relative\;organ\;weight\;\left(\frac{g}{100\;g}\right)=\frac{total\;organ\;weight\times 100}{final\;body\;weight}$$

### 4.4. Analysis of Essential and Toxic Elements by ICP-MS

The concentrations of essential and toxic elements in brain tissue and serum were measured by using ICP-MS and Agilent 7500cx apparatus (Agilent Technologies, Waldbronn, Germany). Prior to use, all glassware and plasticware were cleaned by soaking in diluted HNO_3_ (5%, *v*/*v*) and rinsed with ultrapure water. The sample processing protocol was optimized and modified according to the protocol by Odeh et al. [55]. All samples were digested in closed vessels using a microwave oven decomposition system (Multiwave 3000, Anton Paar, Graz, Austria), according to the procedures outlined by Odeh et al. [55]. The ICP-MS working parameters and conditions were previously described by Babić Leko et al. [56]. All measurements were performed in triplicate for each sample.

Measuring parameters and instrument sensitivity were set before each ICP-MS analysis. For ICP-MS calibration, certified multi-element standards with 99.99% purity for all elements were used (concentration 10 mg/L, composed of aluminum, iron, and calcium; Environmental Calibration Standard, Agilent Technologies, Santa Clara, CA, USA). Stock solutions were prepared by diluting the multi-element standard mixture with ultrapure water (resistivity 18.2 MΩ·cm) obtained from the Milli-Q Advantage A10 system (Millipore Corporation Merck, Darmstadt, Germany). Stock solutions were further diluted with 5% (*v*/*v*) HNO_3_ (Merck, Darmstadt, Germany) by serial dilution to prepare solutions, which were kept at room temperature until use.

The ICP-MS system included a quartz cyclonic spray chamber and a Meinhard^®^ nebulizer connected to the spectrometer’s peristaltic pump via Tygon^®^ tubes. The ICP-MS operated with platinum sampler and skimmer cones. The peristaltic pump was set at 0.40 rps. High-purity argon (99.999%, White Martins, Rio de Janeiro, Brazil) was used throughout [57]. Commercially available reference materials were used to test the accuracy and precision of the ICP-MS method: bovine liver 1577b (NIST, Gaithersburg, MD, USA), equine kidney H8 (IAEA, Vienna, Austria), DORM-2 marine muscle (NRCC, Ottawa, ON, Canada), animal bone H5 (IAEA), swine kidney BCR-186R (IRMM, Geel, Belgium), Seronorm Trace Elements Serum Level I and II (Sero AS, Billingstad, Norway), and plasma ClinChek Control Level I and II (RECIPE, Munich, Germany).

### 4.5. Tissue Isolation and Preparation

Isolated brain tissues were stored at −80 °C until preparation for analysis. After one week, 75–90 mg of tissue was weighed into an Eppendorf tube, and phosphate-buffered saline (PBS, pH 7.4) (Biognost, Zagreb, Croatia) was added at a 1:10 ratio. Samples were then homogenized using an ultrasonic homogenizer (SONOPLUS Bandelin HD2070, Bandelin Electronic GmbH & Co. KG, Berlin, Germany) equipped with a probe MS73 (Bandelin). Homogenization was performed on ice for 30 s in three 10-s intervals. The samples were then centrifuged (ultracentrifuge Mikro 200R, Hettich, Germany) at 15,000 rpm for 10 min at 4 °C.

Before analyzing OS markers (MDA, SOD, CAT, GSH), BDNF, AChE activity, and pro-inflammatory cytokines and chemokines) in brain tissue samples, the supernatant was separated into clean Eppendorf tubes and diluted 10-fold with PBS (100 μL supernatant and 900 μL phosphate buffer). All samples were stored at −80 °C until analysis.

### 4.6. Assessment of OS Markers in Brain Tissue

Brain tissue samples were used to evaluate the redox status through indirect methods for the detection of ROS, i.e., by measuring oxidative damage to lipids and proteins. The total protein content in brain tissues was determined by the Lowry method [58], with bovine serum albumin (BSA) as the standard. The concentrations of OS markers (MDA, CAT, total GSH, and SOD) were normalized to protein levels.

#### 4.6.1. Protein Carbonyl Content in Brain Tissues

The protein carbonyl (PC) content, a marker for oxidative protein modification, was measured using the Levine method [59]. PCs are generated due to the oxidation of protein backbones and amino acid residues (proline, arginine, lysine, and threonine) by ROS molecules. Oxidized proteins react with 2,4-dinitrophenylhydrazine (DNPH) dissolved in HCl, forming 2,4-dinitrophenylhydrazone, which has an extinction coefficient of 22 mM^−1^ cm^−1^ at 370 nm. The absorbance was measured on the spectrophotometer (Libra S22, Biochrom Ltd., Cambridge, UK) using 2 M HCl as the blank. The concentration of carbonylated proteins was expressed as nmol/mg protein.

#### 4.6.2. Malondialdehyde (MDA) Assay in Brain Tissues

LPO, an indicator of ROS-induced membrane damage, was determined using a modified method described by Jayakumar et al. [60]. MDA, an end-product of polyunsaturated fatty acid peroxidation, was quantified in tissue homogenates based on its reaction with thiobarbituric acid (TBA). MDA reacts with two equivalents of TBA to form the MDA-(TBA)_2_ complex, which was measured spectrophotometrically (Libra S22) at 532 nm. The total MDA concentration was calculated using the extinction coefficient for MDA (ε = 155 mM^−1^ cm^−1^) and expressed as nmol MDA/mg protein.

#### 4.6.3. Total GSH Assay in Brain Tissues

GSH, the most abundant intracellular low-molecular-weight thiol, plays a critical role in metabolic protection, including hydroperoxide reduction, xenobiotic detoxification, and free radical scavenging. GSH levels were determined using a modified method by Tietze [61]. The absorbance of total GSH was measured at 412 nm using a microplate reader (Model 550, Bio-Rad, Hercules, CA, USA). Briefly, 40 μL of 10 mM 5,5′-dithiobis-(2-nitrobenzoic acid) (DTNB, Ellman’s Reagent) was mixed with 20 μL of tissue supernatant pretreated with 40 µL of 0.035 M HCl and incubated for 10 min. DTNB reacts with GSH to form the chromophores 5-thionitrobenzoic acid (TNB) and GS-TNB. GS-TNB is further reduced by glutathione reductase to GSH and more TNB, thus enhancing the sensitivity of the assay. The concentration of reduced GSH was determined using a GSH standard (Sigma-Aldrich, Darmstadt, Germany) and expressed as μM GSH/mL protein.

#### 4.6.4. SOD Activity in Brain Tissues

SOD is an antioxidant enzyme that regulates ROS levels by catalyzing the conversion of superoxide anions into hydrogen peroxide and molecular oxygen. SOD activity was measured using the Flohé and Ötting method [62], based on the inhibition of xanthine oxidation (ΔA/min ≈ 0.025), which produces superoxide anions as a substrate for SOD present in the sample. Briefly, 25 µL of sample was mixed with 1.45 mL of reaction solution (0.05 mM cytochrome c and 1 mM xanthine, mixed in a 10:1 (*v*/*v*) ratio with DTNB). The reaction was monitored over 3 min at 550 nm using a Libra S22 spectrophotometer (Biochrom). One unit of total SOD activity was defined as the amount of enzyme required to achieve 50% inhibition on a standard calibration curve. SOD activity was expressed as U/mg protein.

#### 4.6.5. CAT Activity in Brain Tissues

CAT is an antioxidant enzyme responsible for degrading hydrogen peroxide into water and oxygen. CAT activity was measured spectrophotometrically using the Aebi method [63]. The assay is based on monitoring the decrease in the amount of H_2_O_2_ over time. CAT activity was measured at 240 nm (UV-160, Shimadzu, Kyoto, Japan) for one minute. The activity was calculated using the molar absorption coefficient of H_2_O_2_ (ε = 39.4 mM^−1^ cm^−1^). The specific activity was expressed as U/mg protein, corresponding to the μmol of H_2_O_2_ degraded per minute per mg of protein.

### 4.7. Histopathological Analysis of Brain Changes

After sacrifice, the brain was removed, weighed, washed in 0.9% saline, and fixed in 4% neutral buffered formaldehyde (Kemika d.o.o., Zagreb, Croatia). Following fixation, brain samples were prepared according to the standard paraffin embedding procedure. Sections of 7 microns were stained with hematoxylin-eosin (HE—Kemika d.o.o., Zagreb, Croatia). Neurodegenerative changes were assessed through histological analysis after staining with the modified Bielschowsky method (silver staining) [64] and immunohistochemical labeling of Aβ markers (4G8), neurofibrillary tangles (AT8, PHF1, MC1) and microglia (Iba1, CD68, and HLA-DR). Comparable brain areas were analyzed with a light microscope (Olympus, 21× magnification). To visualize neuropathological changes, sagittal brain sections were subjected to immunohistochemical labeling using: (i) primary antibodies: purified (azide-free) anti-Aβ 17–24 antibody (4G8) diluted 1:2000 (BioLegend, San Diego, CA, USA); anti-phospho-PHF-tau pSer202/Thr205 (AT8) diluted 1:500 (Thermo Fisher Scientific, Waltham, MA, USA); anti-tau pSer396/404 (PHF1) diluted 1:1500; Tau-001 (MC1) diluted 1:20 (provided by Dr. Peter Davies, Albert Einstein College of Medicine, Bronx, NY, USA); CD68 (E-11) diluted 1:1250; HLA-DR diluted 1:300 (Dako, Glostrup, Denmark); and Iba1 diluted 1:250 (Wako Pure Chemical Industries, Osaka, Japan); (ii) secondary antibody HRP from VECTASTAIN^®^ ABC-HRP Kit diluted 1: 200 (Vector Laboratories, Newark, CA, USA); (iii) tertiary antibody VECTASTAIN^®^ ABC Kit, diluted 1: 200 (Vector Laboratories); and (iv) BSA (Sigma Aldrich).

Visualization was performed using diaminobenzidine (3,3′-diaminobenzidine, DAB) (SIGMAFAST DAB with Metal Enhancer Tablet Set, cat. no. D0426), prepared according to the manufacturer’s instructions (Sigma-Aldrich). The slides were spotted with the visualization solution, incubated at room temperature for 10 min, then washed with PBS and distilled water. After drying in the dark, the slides were transferred to Histoclear (Thermo Fisher Scientific). The sections were covered with a drop of Histomount (Poly-Mount, catalog number 08381-120; Polysciences, Warrington, PA, USA) and a coverslip. Microscopic images were captured using a Nikon YS2-H Alphaphot-2 microscope with a Nikon DXM 1200 digital camera and the Nikon ACT-1 program, as well as with an AxioCam ERc5s Zeiss digital camera using the AxioCam ERc5s-ZEN2 program. Quantitative analysis of the images (number, area, and staining intensity) was performed using the Nikon ACT-1 and AxioCam ERc5s-ZEN2 programs.

For the Iba1 marker, a semiquantitative analysis was conducted by assessing immunoreactivity according to the following scale:

0 = no immunoreactivity;

1 = few immunoreactive cells, all branched;

2 = moderate number of immunoreactive cells, mostly branched with some activated;

3 = numerous diffusely distributed immunoreactive cells, all activated;

4 = large clusters of activated microglial cells.

Hippocampal formation areas (GD), hilus, CA2/3, CA1, SUB, and white matter were identified according to the method described by West and Gundersen [65].

### 4.8. Determination of BDNF

BDNF is a member of the neurotrophin family and plays an important role in synaptic plasticity and neuronal survival. It is secreted by neurons and is implicated in various dysfunctions, including depression, cognitive decline, and neurodegenerative disorders, such as AD.

BDNF levels in brain tissue homogenates were quantified using a sandwich enzyme-linked immunosorbent assay (ELISA) kit (Rat BDNF ELISA Kit, Thermo Fisher Scientific) according to the manufacturer’s protocol. The minimum detectable dose of rat BDNF is 12 pg/mL. The BDNF concentration was determined using a standard curve, with absorbance measured at 450 nm on a microplate reader (Model 550, Bio-Rad).

### 4.9. Determination of AChE Activity

AChE activity was measured using the colorimetric method described by Ellman et al. [66]. Enzyme activity was determined by measuring the formation of thiocholine, a by-product of acetylcholine (ACh) hydrolysis. The thiocholine reacts with 5,5′-dithiobis-(2-nitrobenzoic acid) (DTNB), yielding a yellow complex that absorbs at 412 nm (ε = 1.36 × 10^4^ M^−1^cm^−1^). The reaction was performed using 40 μL of clear supernatant in a cuvette containing 1.4 mL of phosphate buffer, 25 μL of DTNB, and 35 μL of acetylthiocholine iodide. Absorbance was then measured at 412 nm for 30 s (UV-160, Shimadzu Corporation, Kyoto, Japan). The AChE activity was calculated and expressed as mol/L/min/g tissue.

### 4.10. Neuroinflammation Examination

AlCl_3_ is a neurotoxin known to induce OS and inflammation. The levels of pro-inflammatory cytokines and chemokines in brain samples were determined using a Multi-Analyte ELISArray Kit (Qiagen, Hilden, Germany). This ELISA kit measures the levels of 12 cytokines and chemokines (IL-1α, IL-1β, IL-2, IL-4, IL-6, IL-10, IL-12, IL-13, INF-γ, TNF-α, GM-CSF, and RANTES.

The assay was performed according to the manufacturer’s instructions, and absorbance was measured at 450 nm with correction at 570 nm using a Labsystems iEMS microtiter reader Reader MF (Labsystem, Karlsruhe, Germany). Cytokine concentrations were calculated using a standard curve and expressed in pg/mL.

### 4.11. Neurological Screening

To identify and assess motor and sensory deficits in rats after administration of test components, a NS was conducted following an adapted protocol from Choen et al. [67]. The evaluation was performed the day before treatment initiation and 24 h after treatment completion. Neurological reflex tests included: (i) righting after being placed on the dorsal side; (ii) eye blinking (response to a light touch at the outer corner of the eye with a thin brush); (iii) ear twitching and limb withdrawal in response to tactile stimuli (light touch with a latex gloved finger); (iv) orienting olfactory response to an orange extract and visual stimulus (flashlight); and (v) startle response to an auditory stimulus (metal clicker). The final assessment of neurological deficits was based on the absence or presence of responses to these stimuli.

### 4.12. Statistical Analysis

Results are reported as the mean value ± standard error mean (mean ± SEM). Data were analyzed using the Kruskal–Wallis ANOVA test. Further analysis of differences between groups was performed by multiple comparisons of the mean values of all groups. The data for essential and toxic elements, cytokines, BDNF, and AChE activity were evaluated using Student’s *t*-test. Statistical analyses were performed using STATISTICA 12 program (StatSoft, Tulsa, OK, USA). Statistical significance was set at the level of *p* ≤ 0.05.

## 5. Conclusions

Summarizing the obtained results, we can confirm that aluminum is an environmental neurotoxin that causes neurodegeneration. Quercetin successfully modulated the impaired homeostatic and neuropathological consequences of Al/D-gal-induced Alzheimer’s disease: it directly protected neurons by regulating the level of oxidative stress by increasing the capacity of antioxidant protection; it reduced Aβ aggregation by inhibiting AChE activity; it had a positive effect on reducing neurohistopathological changes; it prevented cell loss in the CA1 area of the hippocampus and reduced the number of diffuse Aβ plaques in the cerebral cortex, hippocampus, and cerebellum; it increased the survival, growth, and differentiation of nerve cells by maintaining the BDNF level; and it regulated microglial immunoreactivity and neuroinflammation in the cerebral cortex, hippocampus, and cerebellum by partial or complete inhibition of proinflammatory cytokines. Multiple effects confirm that quercetin and foods rich in quercetin can be applied as an alternative non-pharmaceutical approach in reducing Al-induced neurotoxicity and maintaining adaptive homeostasis, which consequently affects the functioning of the CNS and the whole organism.

## Figures and Tables

**Table 1:** Toxic and essential elements in serum and brain tissue after chronic administration of aluminum chloride + D-galactose and quercetin.

Serum ^a^	Al (ppm)	Ca (ppm)	Cu (ppm)	Fe (ppm)
**HC**	2.93 ±0.63	81.76 ±1.74	864.02 ± 19.34	3.37 ± 0.22
**AD**	166.10 ± 27.71 **	85.33 ± 0.44	1236.00 ± 118.59 *	2.75 ± 0.07 *
**AD + Qu_25_**	174.89 ± 5.97 ****	82.42 ± 0.47 ^ΔΔ^	1342.14 ± 86.52 **	2.03 ± 0.11 **^ΔΔ^
**AD + Qu_50_**	241.05 ± 23.58 ****	84.36 ± 1.43	1373.25 ± 75.83 **	1.92 ± 0.21 **^Δ^
**Qu_25_**	5.18 ± 0.14 *	86.89 ± 0.31 *	1261.92 ± 113.19 *	3.54 ± 0.28
**Qu_50_**	4.57 ± 1.11	86.75 ± 1.47	1292.22 ± 105.43 *	3.27 ± 0.37
**Brain tissue ^a^**				
**HC**	0.07 ± 0.004	51.83 ± 2.12	3.01 ± 0.05	23.05 ± 0.35
**AD**	0.24 ± 0.016 ***	550.13 ± 39.01 ***	3.14 ± 0.07	24.35 ± 0.41 *
**AD + Qu_25_**	0.19 ± 0.008 *	406.67 ± 13.78 **^Δ^	3.08 ± 0.05	23.00 ± 0.37 ^Δ^
**AD + Qu_50_**	0.14 ± 0.008	264.97 ± 5.70 *^ΔΔ^	3.06 ± 0.15	22.30 ± 0.42 ^ΔΔ^
**Qu_25_**	0.08 ± 0.004	83.49 ± 2.25	3.03 ± 0.15	23.25 ± 0.52
**Qu_50_**	0.07 ± 0.004	65.021 ± 8.43	3.02 ± 0.04	23.37 ± 0.17

^a^ The rats (n = 3 per group) were injected with aluminum chloride + D-galactose (10 mg/kg + 60 mg/kg) and quercetin at doses of 25 or 50 mg/kg through the intraperitoneal route (*i.p.*) for 28 days. Control rats were injected *i.p.* with saline solution. Data are expressed as the mean ± SEM and analyzed using Student’s *t*-test. * Significantly different compared to HC (* *p* ≤ 0.05; ** *p* ≤ 0.01, *** *p* ≤ 0.001; **** *p* ≤ 0.0001). ^Δ^ Significantly different compared to the AD model (^Δ^ *p* ≤ 0.05; ^ΔΔ^ *p* ≤ 0.01). Abbreviations: HC—Healthy control group; AD—Alzheimer’s disease rat model; AD + Qu_25_—AD model injected with quercetin *i.p.* at a dose of 25 mg/kg; AD + Qu_50_—AD model injected with quercetin *i.p.* at a dose of 50 mg/kg; SEM—standard error of the mean.

**Table 2:** Semiquantitative expression of the Iba1 marker in the cerebral cortex and hippocampal formation of the rat brain following chronic administration of aluminum chloride + D-galactose and quercetin.

Experimental Groups	Ctx	Hpp		
		GD	CA1	SUB
**HC**	0	0	2	3
**AD**	3	4	3	4
**AD + Qu_25_**	0	2	2	1
**AD + Qu_50_**	1	1	0	1
**Qu_25_**	0	1	0	0
**Qu_50_**	0	0	0	0

The rats (n = 6 per group) were injected with aluminum chloride + D-galactose (10 mg/kg + 60 mg/kg) and quercetin at a dose of 25 or 50 mg/kg through the intraperitoneal route (*i.p.*) for 28 days. Control rats were injected *i.p.* with saline solution. Abbreviations: HC—Healthy control group; AD—Alzheimer’s disease rat model; AD + Qu_25_—AD model injected with quercetin *i.p.* at a dose of 25 mg/kg; AD + Qu_50_—AD model injected with quercetin *i.p.* at a dose of 50 mg/kg; Ctx—cortex, Hpp—hippocampal formation (GD—gyrus dentatus, CA1—cornu ammonis and SUB—subiculum.

**Table 3:** Cytokine concentrations in brain tissue samples after chronic administration of aluminum chloride + D-galactose and quercetin.

Cytokines	HC	AD	AD + Qu_25_	AD + Qu_50_	Qu_25_	QU_50_
**IL-1α (pg/mL)**	2.30 ± 0.02	4.22 ±0.07 **	3.39 ± 0.06	2.76 ± 0.02	2.55 ± 0.03	2.8 ± 0.02
**IL-1β (pg/mL)**	79.15 ± 0.03	168.25 ± 0.13 **	149.16 ± 0.2	118.9 ± 0.04 ^Δ^	91.89 ± 0.06	117.4 ± 0.1
**IL-2 (pg/mL)**	6.30 ± 0.03	11.25 ± 0.10 **	10 ± 0.15	8.81 ± 0.03 ^Δ^	6.45 ± 0.01	7.71 0.02
**IL-4 (pg/mL)**	0.86 ± 0.03	1.59 ± 0.02 *	1.51 ± 0.03 *	1.14 ± 0.04	0.95 ± 0.02	1.14 ± 0.02
**IL-6 (pg/mL)**	23.72 ± 0.2	51.36 ± 0.30 **	43.57 ± 0.16	33.23 ± 0.2 ^Δ^	25.36 ± 0.13	31.73 ± 0.06
**IL-10 (pg/mL)**	103.34 ± 0.2	243.54 ± 0.20 **	194.29 ± 0.15	146.67 ± 0.4 ^Δ^	78.45 ± 0.03	69.81 ± 0.07 *
**IL-12 (pg/mL)**	2.90 ± 0.03	5.09 ± 0.06 **	4.16 ± 0.10	3.47 ± 0.18	2.97 ± 0.02	4.27 ± 0.03
**IL-13 (pg/mL)**	17.24 ± 0.08	34.19 ± 0.06 **	28.66 ± 0.20	21.60 ± 0.07	17.53 ± 0.05	23.50 ± 0.10
**IFN-γ (pg/mL)**	18.70 ± 0.2	45.87 ± 0.06 **	42.09 ± 0.05	28.48 ± 0.18	23.88 ± 0.06	32.15 ± 0.03
**TNF-α (pg/mL)**	2.31 ± 0.04	4.60 ± 0.20 **	4.01 ± 0.01	2.95 ± 0.04 ^Δ^	2.87 ± 0.03	3.27 ± 0.09
**GM-CSF (pg/mL)**	1.45 ± 0.04	2.70 ± 0.08 **	2.31 ± 0.06	1.93 ± 0.02	1.54 ± 0.04	1.80 ± 0.03
**RANTES (pg/mL)**	1.74 ± 0.02	2.95 ± 0.10	3.13 ± 0.10 *	2.47 ± 0.05	1.96 ± 0.01	2.30 ± 0.05

The rats (n = 3 per group) were injected with aluminum chloride + D-galactose (10 mg/kg + 60 mg/kg) and quercetin at doses of 25 or 50 mg/kg through the intraperitoneal route (*i.p.*) for 28 days. Control rats were injected *i.p.* with saline solution. Data are expressed as the mean ± SEM and analyzed using the Student *t*-test. * Significantly different compared to the HC group (** *p* ≤ 0.01). ^Δ^ Significantly different compared to the AD model (^Δ^ *p* ≤ 0.05). Abbreviations: HC—Healthy control group; AD—Alzheimer’s disease rat model; AD + Qu_25_—AD model injected with quercetin *i.p.* at a dose of 25 mg/kg; AD + Qu_50_—AD model injected with quercetin *i.p.* at a dose of 50 mg/kg; SEM—standard error mean; IL—interleukin, IFN—interferon, TNF—tumor necrosis factor, GM-CSF—granulocyte-macrophage colony stimulating factor, RANTES—CC chemokine regulated on activation, normal T-cell expressed and secreted.

**Table 4:** Assessment of motor and sensory perception in rats after chronic administration of aluminum chloride + D-galactose and quercetin.

Experimental Groups	Uprightness	Pulling Limbs	Ear Twitch	Blink	Visual Orientation	Auditory Orientation	Olfactory Orientation
**HC**	+/+	+/+	+/+	+/+	+/+	+/+	+/+
**AD**	+/+	+/+	+/+	+/+	+/+	+/+	+/+
**AD + Qu_25_**	+/+	+/+	+/+	+/+	+/+	+/+	+/+
**AD + Qu_50_**	+/+	+/+	+/+	+/+	+/+	+/+	+/+
**Qu_25_**	+/+	+/+	+/+	+/+	+/+	+/+	+/+
**Qu_50_**	+/+	+/+	+/+	+/+	+/+	+/+	+/+

The rats (n = 3 per group) were injected with aluminum chloride + D-galactose (10 mg/kg + 60 mg/kg) and quercetin at doses of 25 or 50 mg/kg through the intraperitoneal route (*i.p.*) for 28 days. Control rats were injected *i.p.* with saline solution. Abbreviations: HC—Healthy control group; AD—Alzheimer’s disease rat model; AD + Qu_25_—AD model injected with quercetin *i.p.* at a dose of 25 mg/kg; AD + Qu_50_—AD model injected with quercetin *i.p.* at a dose of 50 mg/kg; (+)—positive response to stimulus.

**Table 5:** Experimental design showing the grouping, treatment regimens, and administration routes used for the induction of Alzheimer’s disease changes and quercetin interventions in Y59 rats.

Rats Y59	0.9% NaCl	AlCl_3_ (mg/kg)	D-gal (mg/kg)	Quercetin (mg/kg)	Application	Days
**HC**	0.5	-	-	-	*i.p.*	28
**AD**	0.5	10	60	-	*i.p.*	28
**AD +Qu_25_**	0.5	10	60	25	*i.p.*	28
**AD + Qu_50_**	0.5	10	60	50	*i.p.*	28
**Qu_25_**	0.5	-	-	25	*i.p.*	28
**Qu_50_**	0.5	-	-	50	*i.p.*	28

Animals were injected intraperitoneally (*i.p.*) once daily for 28 days. The AD model was induced by *i.p.* injection of aluminum chloride (10 mg/kg) and D-galactose (60 mg/kg). Quercetin was administered at doses of either 25 mg/kg or 50 mg/kg. Control rats received *i.p.* injections of saline solution (0.9% NaCl). Abbreviations: HC—Healthy control group; AD—Alzheimer’s disease rat model; AD + Qu_25_—AD model treated with quercetin (25 mg/kg, *i.p.*); AD + Qu_50_—AD model treated with quercetin (50 mg/kg, *i.p.*); Qu_25_—rats treated with quercetin (25 mg/kg, *i.p.*); Qu_50_—rats treated with quercetin (50 mg/kg, *i.p.*).

## Data Availability

The original contributions generated for this study are included in the article; further inquiries can be directed to the corresponding author.

## Abbreviations

The following abbreviations are used in this manuscript:

AChE	Acetylcholinesterase
AD	Alzheimer’s disease
AGEs	Advanced glycation end products
APP	Amyloid precursor protein
APs	Amyloid plaques
Aβ	Amyloid-beta
BBB	Blood–brain barrier
BDNF	Brain-derived neurotrophic factor
CA1	Cornu Ammonis 1, area of the hippocampus
CAT	Catalase
CNS	Central nervous system
CP	Carbonylated proteins
Ctx	Cerebral cortex
D-gal	D-galactose
GD	Dentate gyrus (Gyrus dentatus)
GM-CSF	Granulocyte-macrophage colony stimulating factor
GSH	Glutathione
H_2_O_2_	Hydrogen peroxide
HC	Healthy control
ICP-MS	Inductively coupled plasma-mass spectrometry
IF	Interferon
IL	Interleukin
LPO	Lipid peroxidation
MAPs	Microtubule-associated proteins
MDA	Malondialdehyde
NFTs	Neurofibrillary tangles
NS	Neurological screening
OS	Oxidative stress
p-tau	Hyperphosphorylated tau
Qu	Quercetin
O_2_˙^−^	Superoxide anion
OH˙	Hydroxyl radical
OH^−^	Hydroxyl anion
PC	Proteins carbonylated
RAGE	Receptors for AGEs
RANTES	Chemokine regulated on activation, normal T-cell expressed and secreted
ROS	Reactive oxygen species
SOD	Superoxide dismutase
SUB	Subiculum
TNF-α	Tumor necrosis alpha (TNF-α)

## References

[1] Zvěřová M. (2019). Clinical aspects of Alzheimer’s disease. Clin. Biochem..

[2] Moya-Alvarado G., Gershoni-Emek N., Perlson E., Bronfman F.C. (2016). Neurodegeneration and Alzheimer’s disease (AD). What Can Proteomics Tell Us About the Alzheimer’s Brain?. Mol. Cell. Proteom..

[3] Hardy J., Orr H. (2006). The genetics of neurodegenerative diseases. J. Neurochem..

[4] Mahdi O., Baharuldin M.T.H., Mohd Nor N.H., Chiroma S.M., Jagadeesan S., Moklas M.A.M. (2019). Chemicals used for the induction of Alzheimer’s disease-like cognitive dysfunctions in rodents. Biomed. Res. Ther..

[5] Kaur R., Sood A., Lang D.K., Bhatia S., Al-Harrasi A., Aleya L., Behl T. (2022). Potential of flavonoids as anti-Alzheimer’s agents: Bench to bedside. Environ. Sci. Pollut. Res. Int..

[6] Igarashi K.M. (2023). Entorhinal cortex dysfunction in Alzheimer’s disease. Trends Neurosci..

[7] Agarwal M., Alam M.R., Haider M.K., Malik M.Z., Kim D.K. (2020). Alzheimer’s Disease: An Overview of Major Hypotheses and Therapeutic Options in Nanotechnology. Nanomaterials.

[8] Chen X., Holtzman D.M. (2022). Emerging roles of innate and adaptive immunity in Alzheimer’s disease. Immunity.

[9] Bharathi V.P., Govindaraju M., Palanisamy A.P., Sambamurti K., Rao K.S. (2008). Molecular toxicity of aluminium in relation to neurodegeneration. Ind. J. Med. Res..

[10] Page K.E., White K.N., McCrohan C.R., Killilea D.W., Lithgow G.J. (2012). Aluminium exposure disrupts elemental homeostasis in Caenorhabditis elegans. Metallomics.

[11] Chiroma S.M., Mohd Moklas M.A., Mat Taib C.N., Baharuldin M.T.H., Amon Z. (2018). D-galactose and aluminium chloride induced rat model with cognitive impairments. Biomed. Pharmacother..

[12] Chen Y., Tian X., Wang X. (2018). Advances in dialysis encephalopathy research: A review. Neurol. Sci..

[13] Jackson J.S., Rout P. (2025). Aluminum Toxicity. StatPearls.

[14] Oršolić N., Jazvinšćak Jembrek M. (2022). Molecular and Cellular Mechanisms of Propolis and Its Polyphenolic Compounds against Cancer. Int. J. Mol. Sci..

[15] Sharma D.R., Wani W.Y., Sunkaria A., Kandimalla R.J., Sharma R.K., Verma D., Bal A., Gill K.D. (2016). Quercetin attenuates neuronal death against aluminum-induced neurodegeneration in the rat hippocampus. Neuroscience.

[16] Costa L.G., Garrick J.M., Roquè P.J., Pellacani C. (2016). Mechanisms of Neuroprotection by Quercetin: Counteracting Oxidative Stress and More. Oxid. Med. Cell. Longev..

[17] Khan A., Ali T., Rehman S.U., Khan M.S., Alam S.I., Ikram M., Muhammad T., Saeed K., Badshah H., Kim M.O. (2018). Neuroprotective Effect of Quercetin Against the Detrimental Effects of LPS in the Adult Mouse Brain. Front. Pharmacol..

[18] Jadhav R., Kulkarni Y.A. (2023). Neuroprotective Effect of Quercetin and Memantine against AlCl3-Induced Neurotoxicity in Albino Wistar Rats. Molecules.

[19] Rahimzadeh M.R., Rahimzadeh M.R., Kazemi S., Amiri R.J., Pirzadeh M., Moghadamnia A.A. (2022). Aluminum Poisoning with Emphasis on Its Mechanism and Treatment of Intoxication. Emerg. Med. Int..

[20] Wang L. (2018). Entry and Deposit of Aluminum in the Brain. Adv. Exp. Med. Biol..

[21] Anyachor C.P., Orish C.N., Ezejiofor A.N., Cirovic A., Cirovic A., Ezealisiji K.M., Orisakwe O.E. (2023). Nickel and aluminium mixture elicit memory impairment by activation of oxidative stress, COX-2, and diminution of AChE, BDNF and NGF levels in cerebral cortex and hippocampus of male albino rats. Curr. Res. Toxicol..

[22] Anyanwu G.E., Nwachukwu I.J., Oria S.R., Obasi K.K., Ekwueme E.P., Nto J.N., Anyanwu N.C. (2024). Fisetin attenuates AlCl_3_-induced neurodegeneration by modulating oxidative stress and inflammatory cytokine release in adult albino wistar rats. Toxicol. Rep..

[23] Gustavsson A., Norton N., Fast T., Frölich L., Georges J., Holzapfel D., Kirabali T., Krolak-Salmon P., Rossini P.M., Ferretti M.T. (2023). Global estimates on the number of persons across the Alzheimer’s disease continuum. Alzheimer Dement..

[24] Li X., Feng X., Sun X., Hou N., Han F., Liu Y. (2022). Global, regional, and national burden of Alzheimer’s disease and other dementias, 1990–2019. Front. Aging Neurosci..

[25] Bonfiglio R., Scimeca M., Mauriello A. (2023). The impact of aluminum exposure on human health. Arch. Toxicol..

[26] Huat T.J., Camats-Perna J., Newcombe E.A., Valmas N., Kitazawa M., Medeiros R. (2019). Metal Toxicity Links to Alzheimer’s Disease and Neuroinflammation. J. Mol. Biol..

[27] Chiroma S.M., Hidayat Baharuldin M.T., Mat Taib C.N., Amom Z., Jagadeesan S., Adenan M.I., Mohd Moklas M.A. (2019). Protective effect of Centella asiatica against D-galactose and aluminium chloride induced rats: Behavioral and ultrastructural approaches. Biomed. Pharmacother..

[28] Jaishankar M., Tseten T., Anbalagan N., Mathew B.B., Beeregowda K.N. (2014). Toxicity, mechanism and health effects of some heavy metals. Interdiscip. Toxicol..

[29] Kaur A., Gill K.D. (2005). Disruption of neuronal calcium homeostasis after chronic aluminium toxicity in rats. Basic Clin. Pharmacol. Toxicol..

[30] Kim Y., Olivi L., Cheong J.H., Maertens A., Bressler J.P. (2007). Aluminum stimulates uptake of non-transferrin bound iron and transferrin bound iron in human glial cells. Toxicol. Appl. Pharmacol..

[31] Sirovina D., Orsolić N., Koncić M.Z., Kovacević G., Benković V., Gregorović G. (2013). Quercetin vs chrysin: Effect on liver histopathology in diabetic mice. Hum. Exp. Toxicol..

[32] Al-Otaibi S.S., Arafah M.M., Sharma B., Alhomida A.S., Siddiqi N.J. (2018). Synergistic Effect of Quercetin and *α*-Lipoic Acid on Aluminium Chloride Induced Neurotoxicity in Rats. J. Toxicol..

[33] Ademosun A.O., Oboh G., Bello F., Ayeni P.O. (2016). Antioxidative Properties and Effect of Quercetin and Its Glycosylated Form (Rutin) on Acetylcholinesterase and Butyrylcholinesterase Activities. J. Evid. Based Complement. Altern. Med..

[34] Abdalla F.H., Cardoso A.M., Pereira L.B., Schmatz R., Gonçalves J.F., Stefanello N., Fiorenza A.M., Gutierres J.M., da Silva Serres J.D., Zanini D. (2013). Neuroprotective effect of quercetin in ectoenzymes and acetylcholinesterase activities in cerebral cortex synaptosomes of cadmium-exposed rats. Mol. Cell. Biochem..

[35] Rani V., Verma R., Kumar K., Chawla R. (2022). Role of pro-inflammatory cytokines in Alzheimer’s disease and neuroprotective effects of pegylated self-assembled nanoscaffolds. Curr. Res. Pharmacol. Drug Discov..

[36] Alqarni S.S., Afzal M., Alharbi K.S., Alenezi S.K., Alsahli T.G., Zaidi S., Altyar A.E., Ghaboura N., Kazmi I., Mantargi M.J.S. (2024). Rosiridin Protects Against Aluminum Chloride-Induced Memory Impairment via Modulation of BDNF/NFκB/PI3K/Akt Pathway in Rats. Medicina.

[37] He C., Chen B., Yang H., Zhou X. (2025). The dual role of microglia in Alzheimer’s disease: From immune regulation to pathological progression. Front. Aging Neurosci..

[38] Hendrickx D.A.E., van Eden C.G., Schuurman K.G., Hamann J., Huitinga I. (2017). Staining of HLA-DR, Iba1 and CD68 in human microglia reveals partially overlapping expression depending on cellular morphology and pathology. J. Neuroimmunol..

[39] Chen L., Shen Q., Liu Y., Zhang Y., Sun L., Ma X., Song N., Xie J. (2025). Homeostasis and metabolism of iron and other metal ions in neurodegenerative diseases. Signal Transduct. Target. Ther..

[40] Baj J., Bargieł J., Cabaj J., Skierkowski B., Hunek G., Portincasa P., Flieger J., Smoleń A. (2023). Trace Elements Levels in Major Depressive Disorder-Evaluation of Potential Threats and Possible Therapeutic Approaches. Int. J. Mol. Sci..

[41] Al-Fartusie F.S., Al-Bairmani H.K., Al-Garawi Z.S., Yousif A.H. (2019). Evaluation of Some Trace Elements and Vitamins in Major Depressive Disorder Patients: A Case-Control Study. Biol. Trace Elem. Res..

[42] Corrente G.A., Malacaria L., Beneduci A., Furia E., Marino T., Mazzone G. (2021). Experimental and theoretical study on the coordination properties of quercetin towards aluminum(III), iron(III) and copper(II) in aqueous solution. J. Mol. Liq..

[43] Nday C.M., Drever B.D., Salifoglou T., Platt B. (2010). Aluminium interferes with hippocampal calcium signaling in a species-specific manner. J. Inorg. Biochem..

[44] Ge M., Zhang J., Chen S., Huang Y., Chen W., He L., Zhang Y. (2022). Role of Calcium Homeostasis in Alzheimer’s Disease. Neuropsychiatr. Dis. Treat..

[45] Guan T., Cao C., Hou Y., Li Y., Wei X., Li S., Jia S., Zhao X. (2021). Effects of quercetin on the alterations of serum elements in chronic unpredictable mild stress-induced depressed rats. Biometals.

[46] Xu D., Hu M.J., Wang Y.Q., Cui Y.L. (2019). Antioxidant Activities of Quercetin and Its Complexes for Medicinal Application. Molecules.

[47] West S., Bhugra P. (2015). Emerging drug targets for *Aβ* and tau in Alzheimer’s disease: A systematic review. Br. J. Clin. Pharmacol..

[48] Colucci-D’Amato L., Speranza L., Volpicelli F. (2020). Neurotrophic Factor BDNF, Physiological Functions and Therapeutic Potential in Depression, Neurodegeneration and Brain Cancer. Int. J. Mol. Sci..

[49] Baral S., Pariyar R., Kim J., Lee H.S., Seo J. (2017). Quercetin-3-*O*-glucuronide promotes the proliferation and migration of neural stem cells. Neurobiol. Aging.

[50] Karimipour M., Rahbarghazi R., Tayefi H., Shimia M., Ghanadian M., Mahmoudi J., Bagheri H.S. (2019). Quercetin promotes learning and memory performance concomitantly with neural stem/progenitor cell proliferation and neurogenesis in the adult rat dentate gyrus. Int. J. Dev. Neurosci..

[51] Liu P., Zou D., Yi L., Chen M., Gao Y., Zhou R., Zhang Q., Zhou Y., Zhu J., Chen K. (2015). Quercetin ameliorates hypobaric hypoxia-induced memory impairment through mitochondrial and neuron function adaptation via the PGC-1 alpha pathway. Restor. Neurol. Neurosci..

[52] Chiang M.C., Tsai T.Y., Wang C.J. (2023). The Potential Benefits of Quercetin for Brain Health: A Review of Anti-Inflammatory and Neuroprotective Mechanisms. Int. J. Mol. Sci..

[53] Sudarshan K., Boda A.K., Dogra S., Bose I., Yadav P.N., Aidhen I.S. (2019). Discovery of an isocoumarin analogue that modulates neuronal functions via neurotrophin receptor TrkB. Bioor. Med. Chem. Lett..

[54] Ramanan M., Sinha S., Sudarshan K., Aidhen I.S., Doble M. (2016). Inhibition of the enzymes in the leukotriene and prostaglandin pathways in inflammation by 3-aryl isocoumarins. Eur. J. Med. Chem..

[55] Odeh D., Oršolić N., Adrović E., Bilandžić N., Sedak M., Žarković I., Lesar N., Balta V. (2024). The Impact of the Combined Effect of Inhalation Anesthetics and Iron Dextran on Rats’ Systemic Toxicity. Int. J. Mol. Sci..

[56] Babić Leko M., Mihelčić M., Jurasović J., Nikolac Perković M., Španić E., Sekovanić A., Orct T., Zubčić K., Langer Horvat L., Pleić N. (2022). Heavy Metals and Essential Metals Are Associated with Cerebrospinal Fluid Biomarkers of Alzheimer’s Disease. Int. J. Mol. Sci..

[57] Chen W., Yang Y., Fu K., Zhang D., Wang Z. (2022). Progress in ICP-MS Analysis of Minerals and Heavy Metals in Traditional Medicine. Front. Pharmacol..

[58] Lowry O.H., Rosebrough N.J., Farr A.L., Randall R.J. (1951). Protein measurment with the Folin phenol reagent. J. Biol. Chem..

[59] Levine R.L. (2002). Carbonyl modified proteins in cellular regulation, aging and disease. Free Radic. Biol. Med..

[60] Jayakumar T., Thomas P.A., Geraldine P. (2007). Protective effect of an extract of the oyster mushroom, *Pleurotus ostreatus*. On antioxidants of major organs of aged rats. Exp. Gerontol..

[61] Tietze F. (1969). Enzymic method for quantitative determination of nanogram amounts of total and oxidized glutathione: Applications to mammalian blood and other tissues. Anal. Biochem..

[62] Flohé L., Ötting F. (1971). Superoxide dismutase assays. Meth. Enzymol..

[63] Aebi H. (1984). Catalase in vitro. Methods Enzymol..

[64] Yamamoto T., Hirano A. (1986). A comparative study of modified Bielschowsky, Bodian and thioflavin S stains on Alzheimer’s neurofibrillary tangles. Neuropathol. Appl. Neurobiol..

[65] West M.J., Gundersen H.J.G. (1990). Unbiased stereological estimation of the number of neurons in the human hippocampus. J. Comp. Neurol..

[66] Ellman G.L., Courtney K.D., Andres V., Feather-Stone R.M. (1961). A new and rapid colorimetric determination of acetylcholinesterase activity. Biochem. Pharmacol..

[67] Cohen R.M., Rezai-Zadeh K., Weitz T.M., Rentsendorj A., Gate D., Spivak I., Bholat J., Vasilevko V., Glabe C.G., Breunig J.J. (2013). A transgenic alzheimer rat with plaques, tau pathology, behavioral impairment, oligomeric *Aβ*, and frank neuronal loss. J. Neurosci..

